# Supplementary material for: Anti-Ovarian Cancer Conotoxins Identified from Conus Venom
Source: Molecules. 2022 Oct 5;27(19):6609. doi: 10.3390/molecules27196609 (PMC9573077; doi:10.3390/molecules27196609)
Supplement: Supplementary file 1 [file molecules-27-06609-s001.zip › molecules-1914956-supplementary-A-done.pdf]

Supplemental Material A

Anti-Ovarian Cancer Conotoxins Identified from *Conus* Venom

Shuang Ju [1], Yu Zhang [1], Xijun Guo [1], Qinghui Yan [1], Siyi Liu [1], Bokai Ma [2], Mei Zhang [2], Jiaolin Bao [1], Sulan Luo [1,3] and Ying Fu [1,*]

[1] Key Laboratory of Tropical Biological Resources of Ministry of Education, School of Pharmaceutical Sciences, Hainan University, Haikou 570228, China
[2] Beijing Key Laboratory of Organic Materials Testing Technology & Quality Evaluation, Institute of Analysis and Testing, Beijing Academy of Science and Technology, Beijing 100094, China
[3] Medical School, Guangxi University, Nanning 530004, China

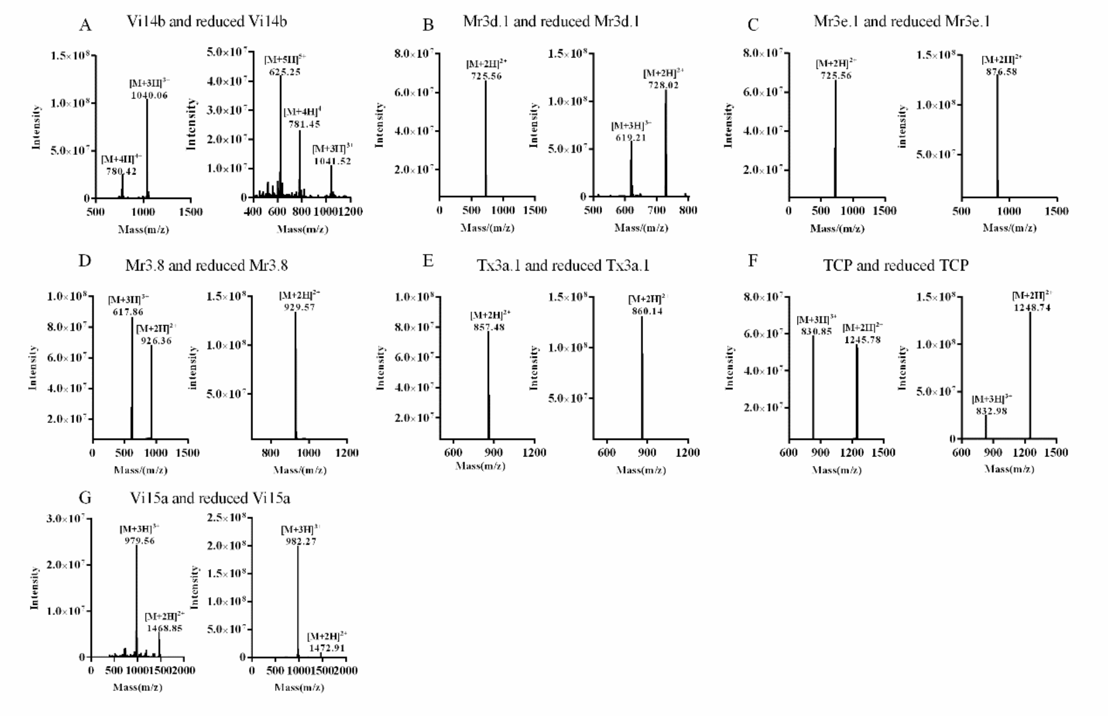

Figure S1. Primary mass spectra of Vi14b, Mr3d.1, Mr3e.1, Mr3.8, Tx3a.1, TCP, Vi15a before and after total reduction.

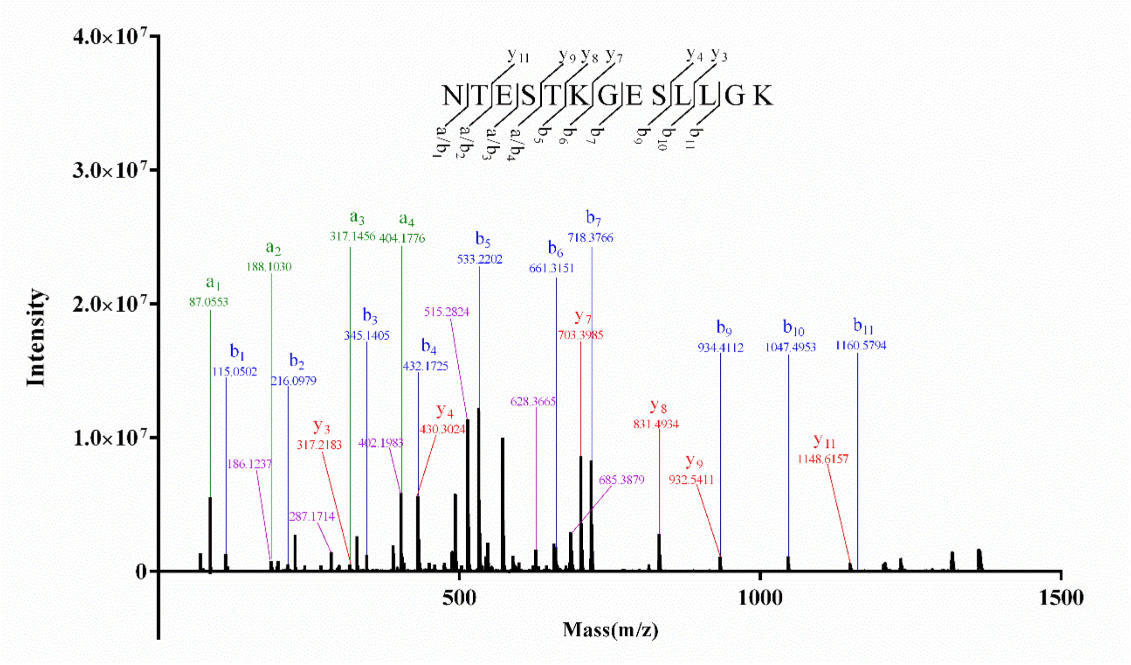

Figure S2. MALDI−TOF−MS/MS spectrum of Vi003 sequencing.

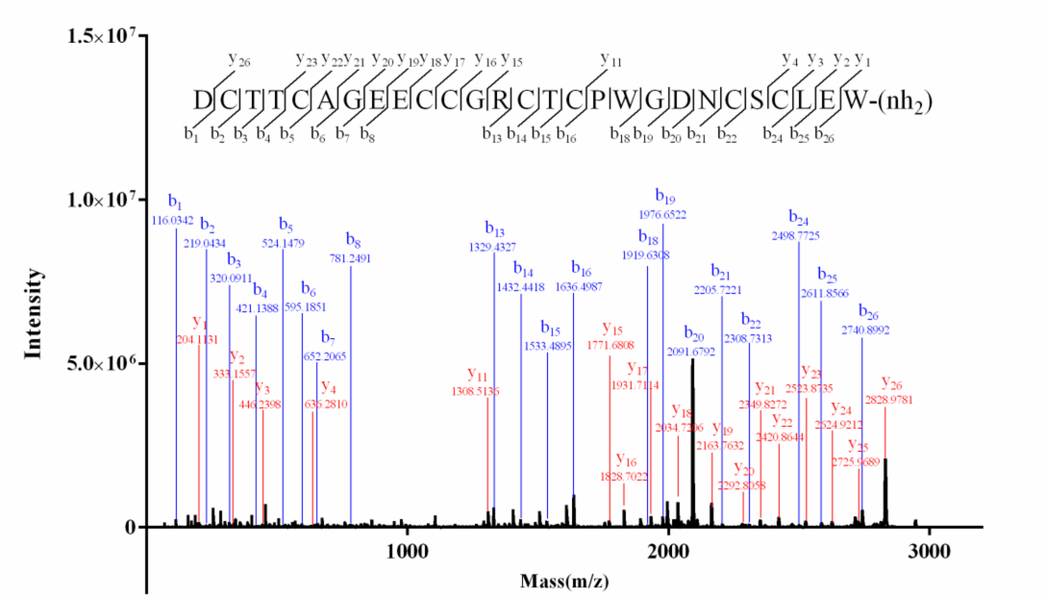

Figure S3. MALDI−TOF−MS/MS spectrum of Vi15a sequencing.

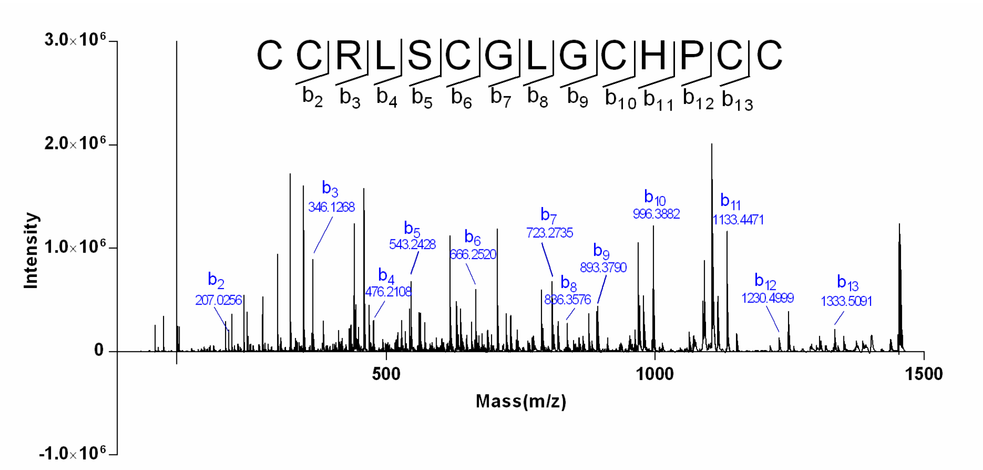

Figure S4. MALDI–TOF–MS/MS spectrum of Mr3d.1 sequencing.

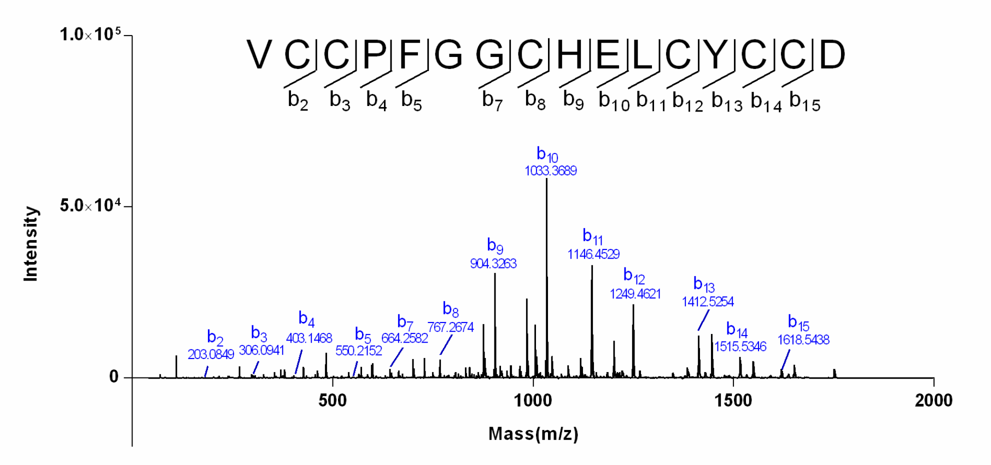

Figure S5. MALDI–TOF–MS/MS spectrum of Mr3e.1 sequencing.

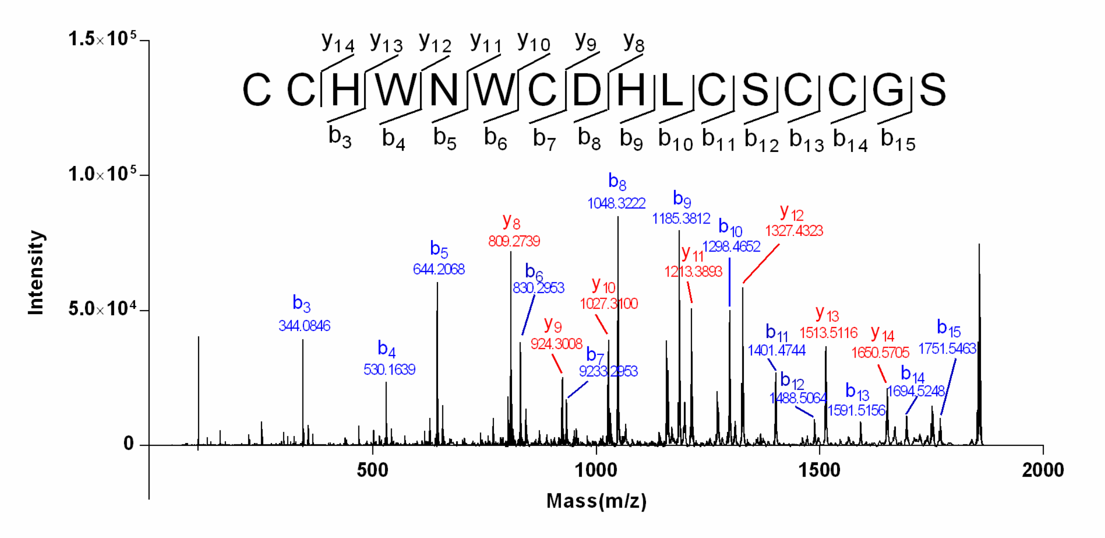

Figure S6. MALDI–TOF–MS/MS spectrum of Mr3.8 sequencing.

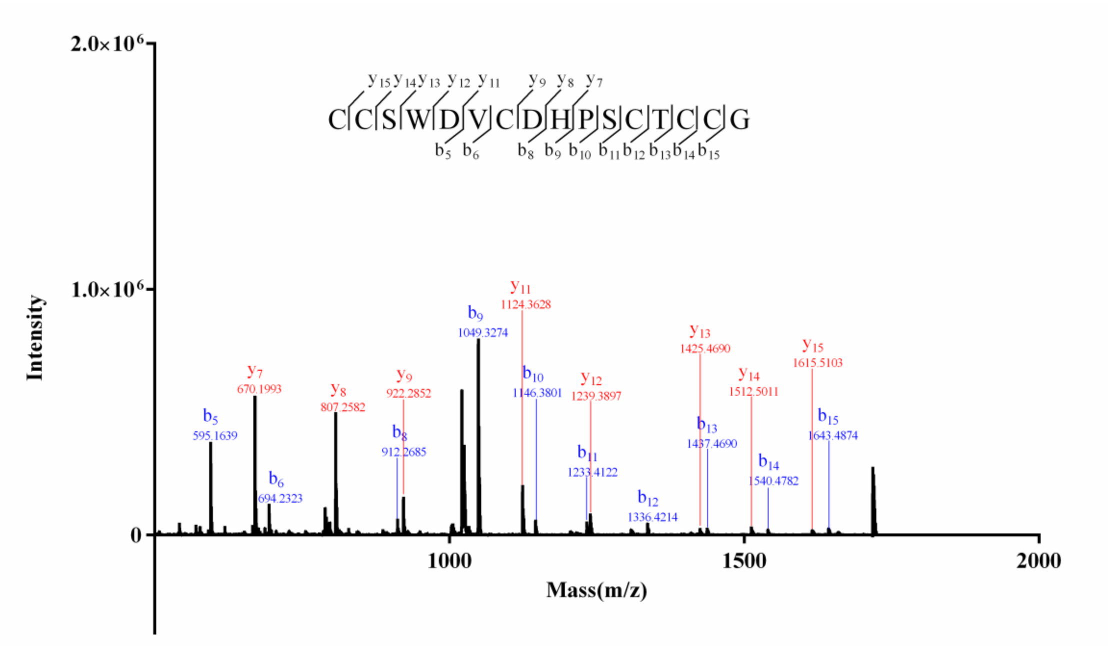

Figure S7. MALDI−TOF−MS/MS spectrum of Tx3a.1 sequencing.

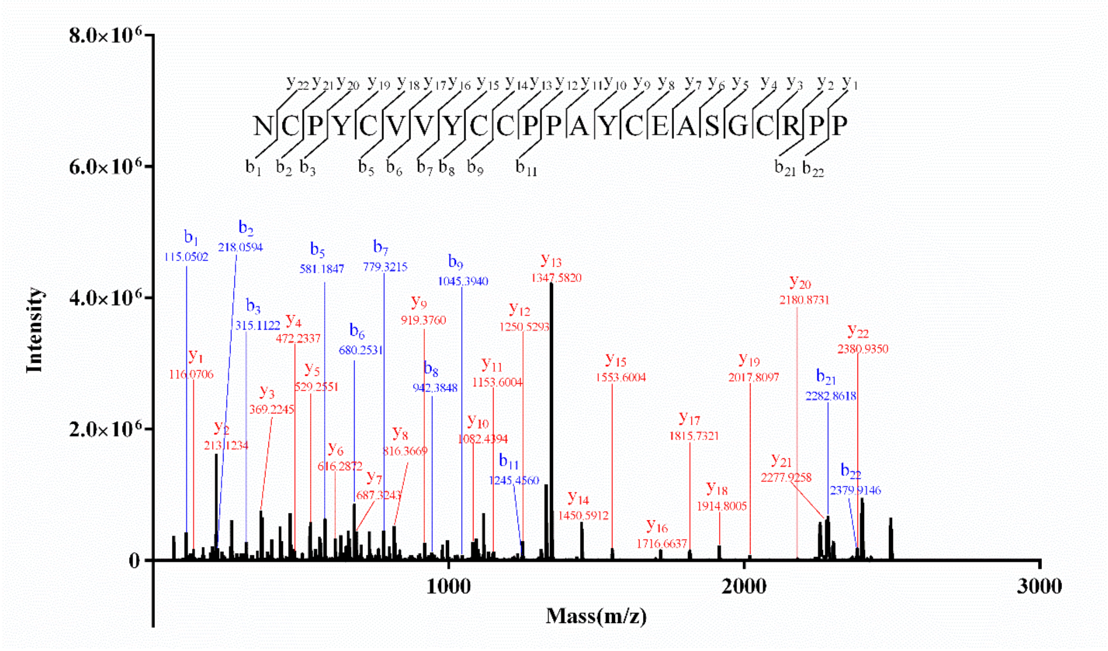

Figure S8. MALDI−TOF−MS/MS spectrum of TCP sequencing.

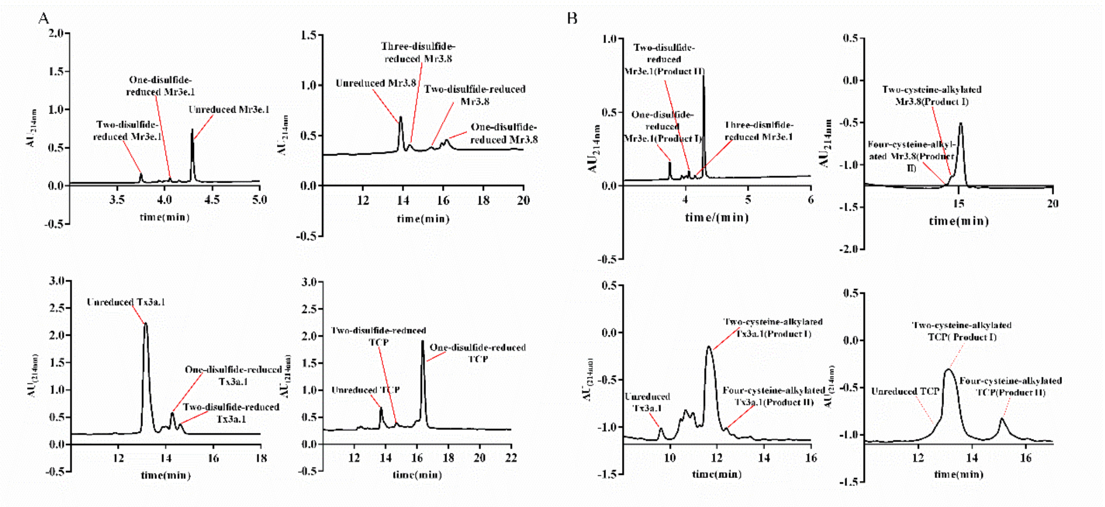

Figure S9. HPLC analysis of stepwise reduction by TCEP and follow-up alkylation by IAM. **(A)** Stepwise reduction products of Mr3e.1, Mr3.8, Tx3a.1 and TCP. **(B)** Alkylated products of stepwise-reduced Mr3e.1, Mr3.8, Tx3a.1 and TCP.

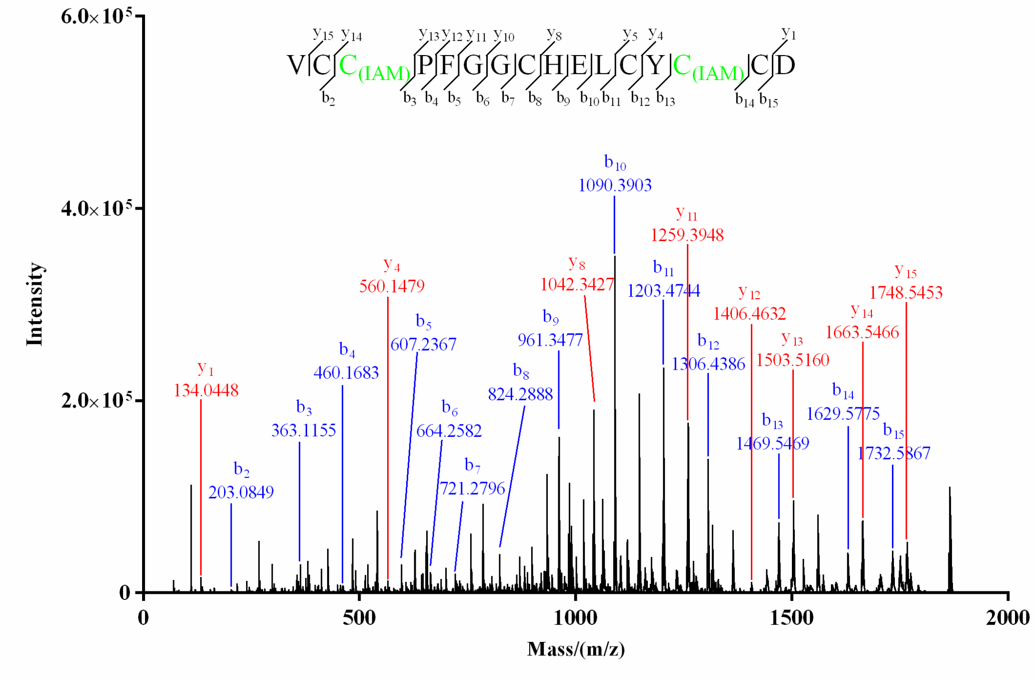

Figure S10. MALDI–TOF–MS/MS spectrum of product I of Mr3e.1.

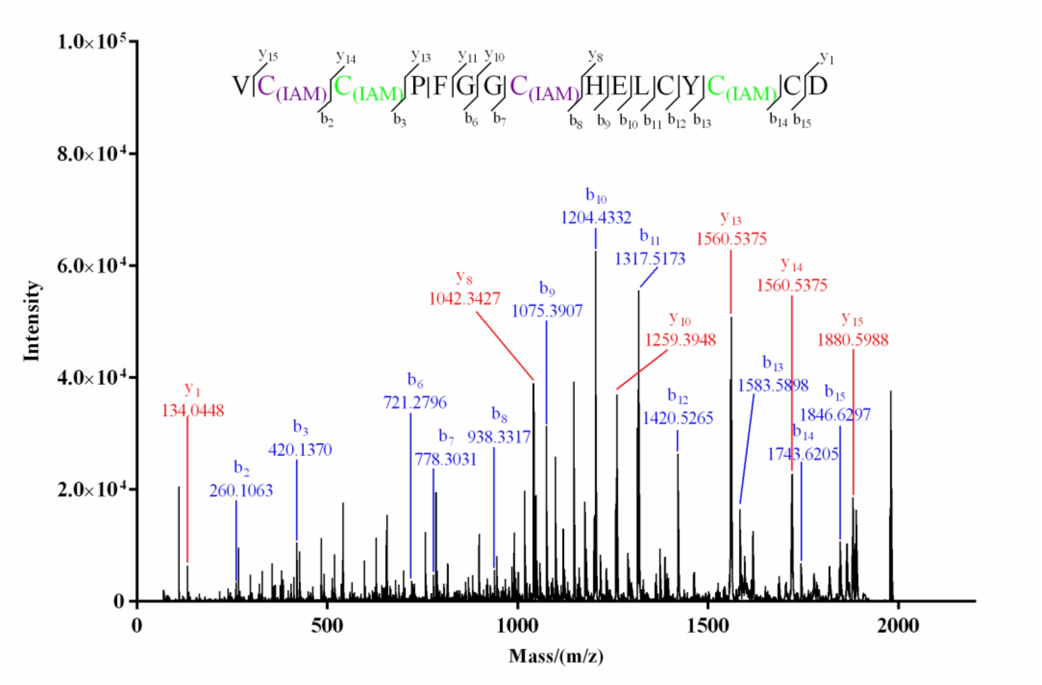

Figure S11. MALDI−TOF−MS/MS spectrum of product II of Mr3e.1.

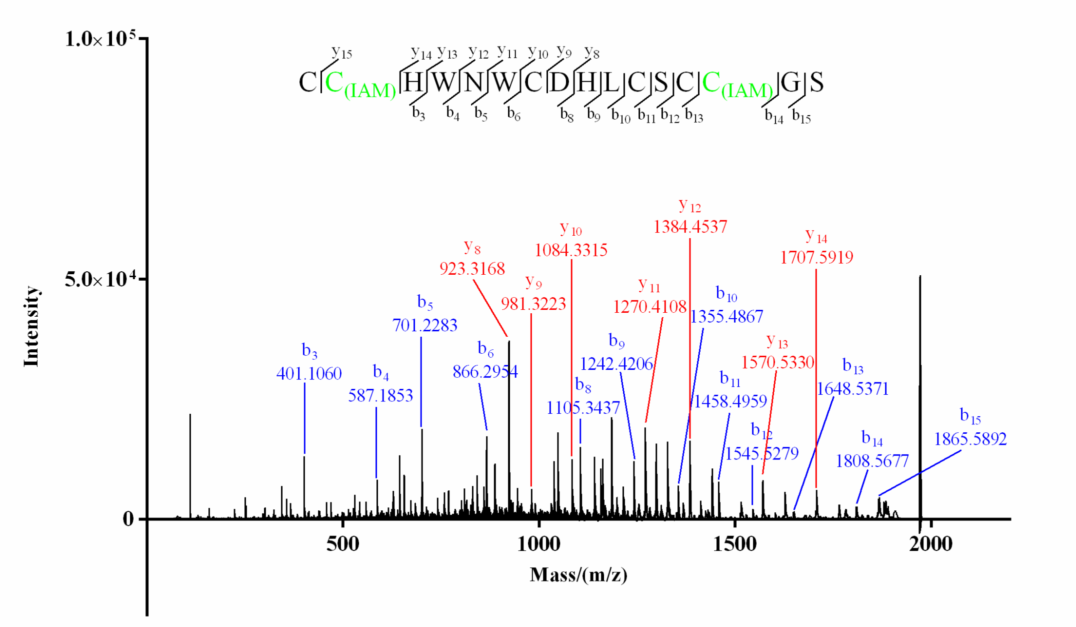

Figure S12. MALDI−TOF−MS/MS spectrum of product I of Mr3.8.

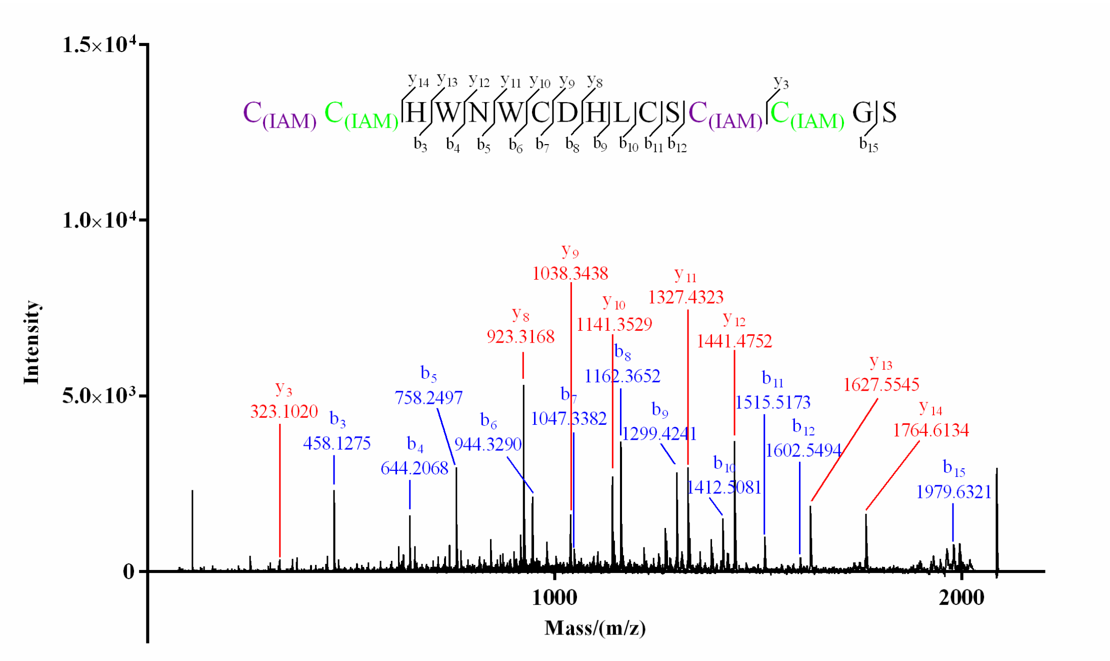

Figure S13. MALDI−TOF−MS/MS spectrum of product II of Mr3.8.

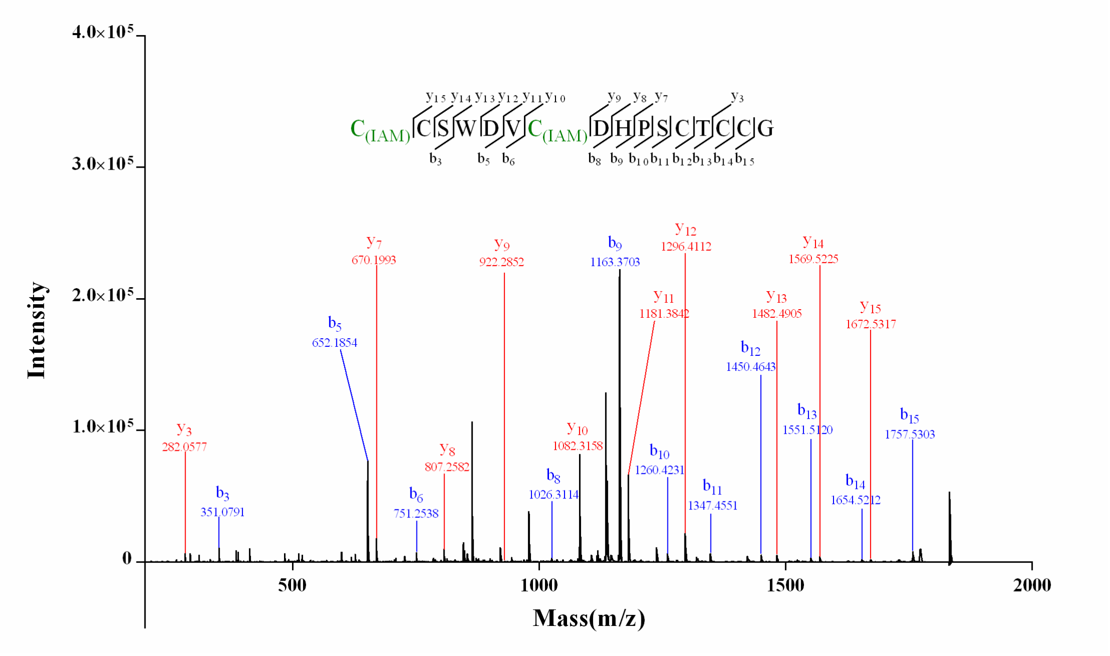

Figure S14. MALDI−TOF−MS/MS spectrum of product I of Tx3a.1.

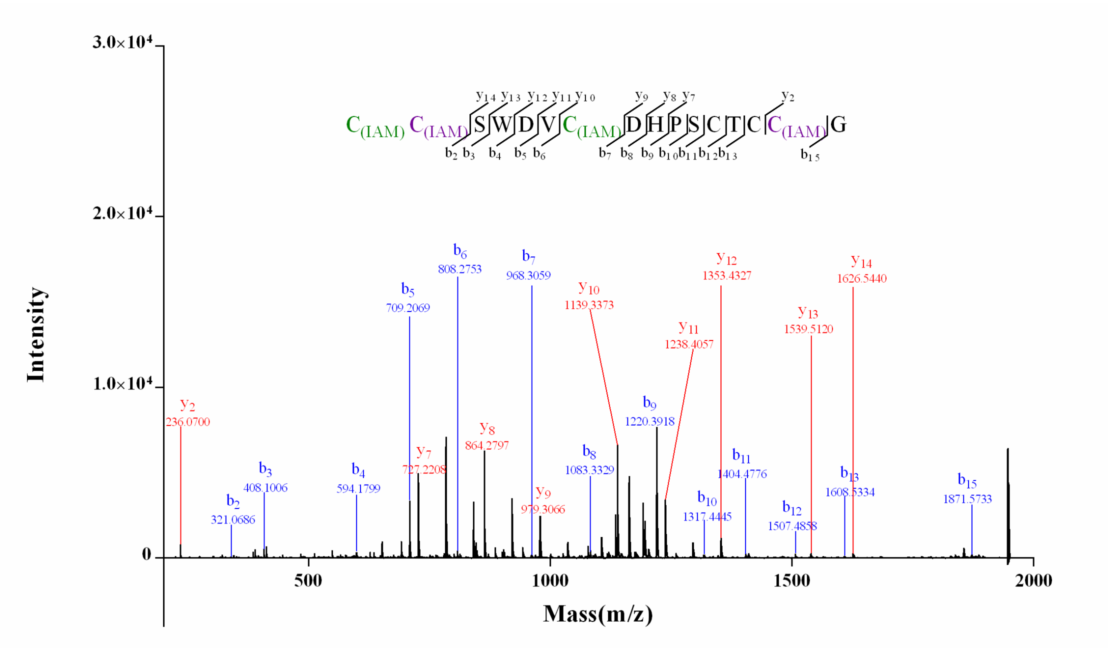

Figure S15. MALDI−TOF−MS/MS spectrum of product II of Tx3a.1.

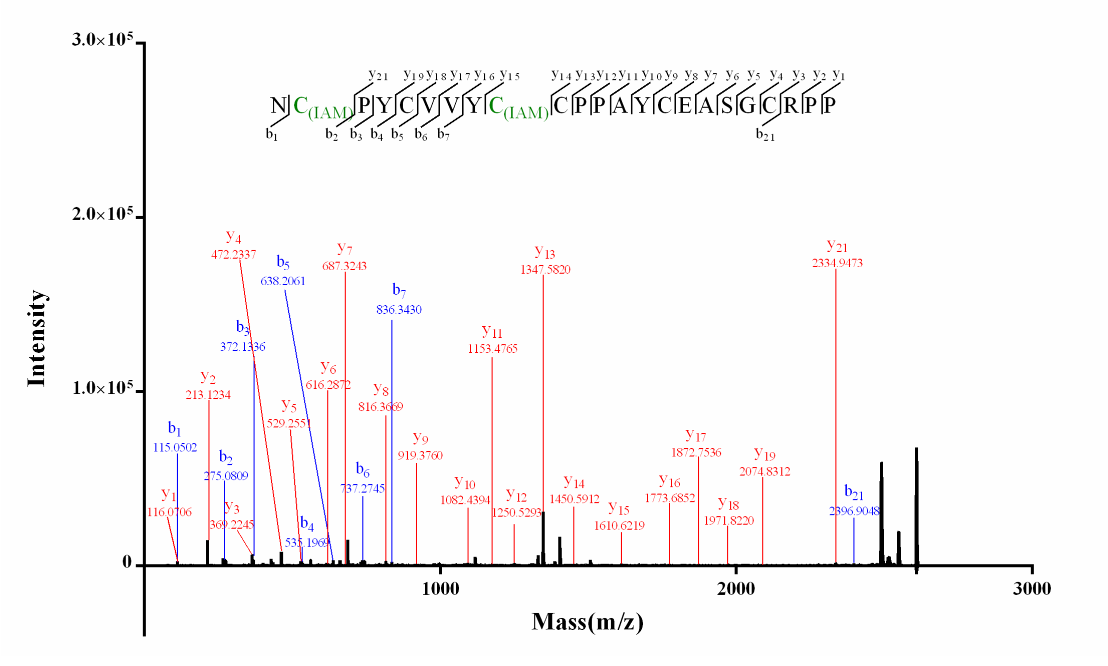

Figure S16. MALDI−TOF−MS/MS spectrum of product I of TCP.

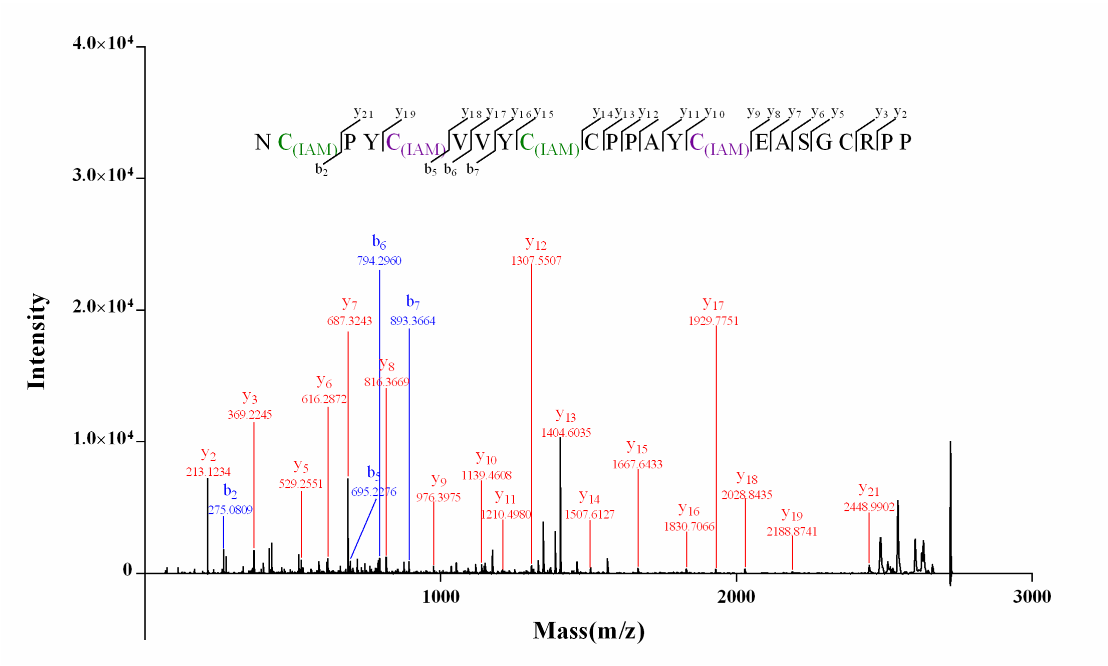

Figure S17. MALDI−TOF−MS/MS spectrum of product II of TCP.
