# Supplementary material for: Anti-Ovarian Cancer Conotoxins Identified from Conus Venom
Source: Molecules. 2022 Oct 5;27(19):6609. doi: 10.3390/molecules27196609 (PMC9573077; doi:10.3390/molecules27196609)
Supplement: Supplementary file 1 [file molecules-27-06609-s001.zip › molecules-1914956-supplementary-B-done.pdf]

Supplemental Material B

Anti-Ovarian Cancer Conotoxins Identified from *Conus* Venom

Shuang Ju [1], Yu Zhang [1], Xijun Guo [1], Qinghui Yan [1], Siyi Liu [1], Bokai Ma [2], Mei Zhang [2], Jiaolin Bao [1], Sulan Luo [1,3] and Ying Fu [1,*]

[1] Key Laboratory of Tropical Biological Resources of Ministry of Education, School of Pharmaceutical Sciences, Hainan University, Haikou 570228, China
[2] Beijing Key Laboratory of Organic Materials Testing Technology & Quality Evaluation,
Institute of Analysis and Testing, Beijing Academy of Science and Technology, Beijing 100094, China
[3] Medical School, Guangxi University, Nanning 530004, China

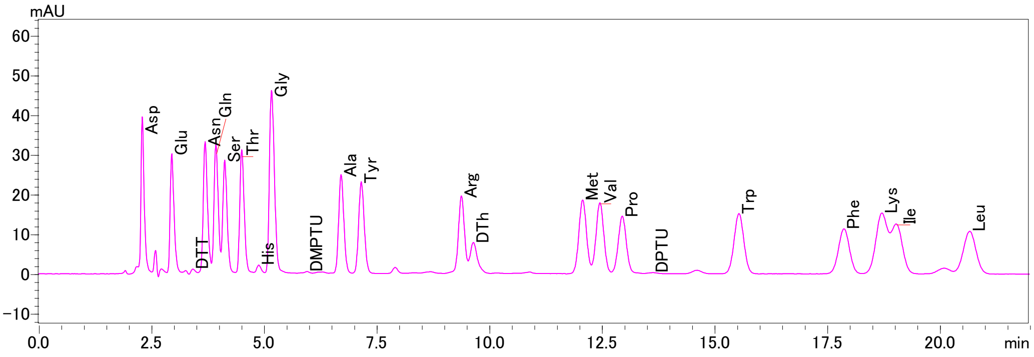

Figure S1. HPLC chromatogram of mixed amino acid standard.

Table S1. Retention time of each amino acid in the HPLC chromatogram of the mixed standard

Constituent	Retention Time (min)
Asp	2.301
Glu	2.951
Asn	3.699
Gln	3.943
Ser	4.122
Thr	4.512
His	4.903
Gly	5.179
Ala	6.708
Tyr	7.163
Arg	9.398
Met	12.091
Val	12.464
Pro	12.980
Trp	15.530
Phe	17.879
Lys	18.653
Ile	19.054
Leu	20.630

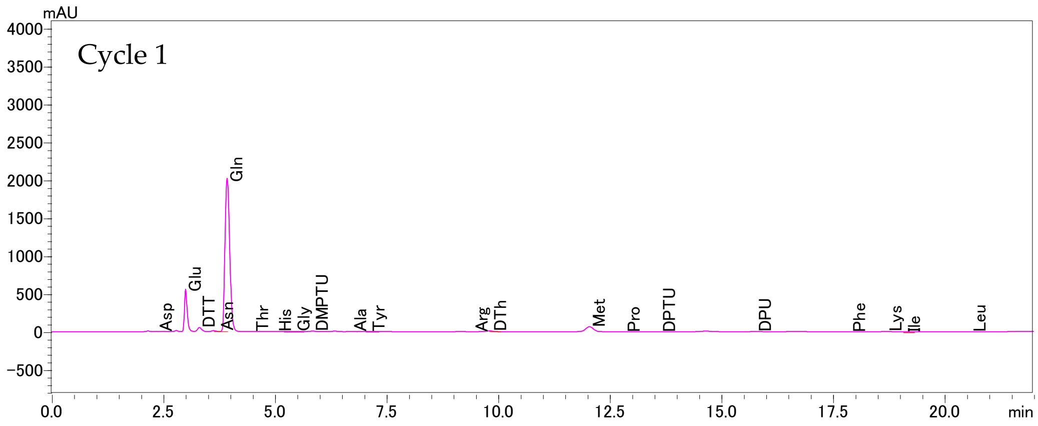

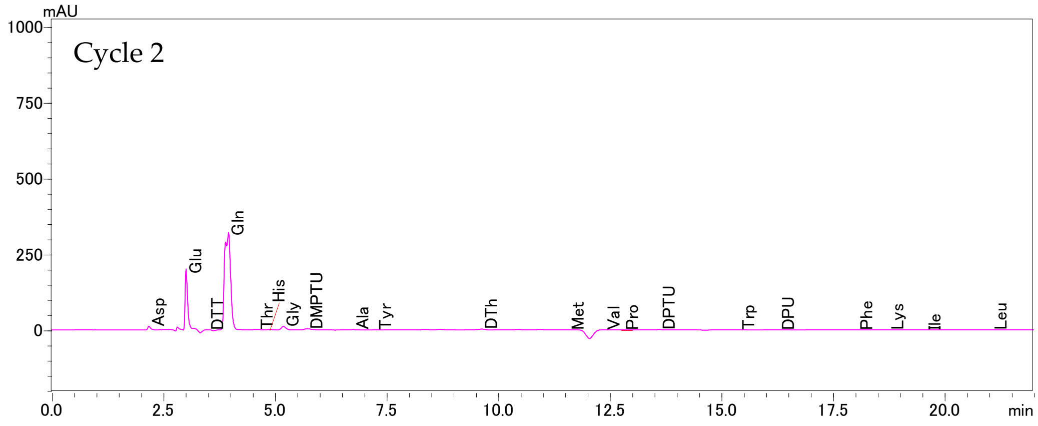
Cycle 2
mAU
1000
750
500
250
0
0.0
2.5
5.0
7.5
10.0
12.5
15.0
17.5
20.0
min
Asp
Glu
DTT
Gln
Thr
His
Gly
DMPTU
Ala
Tyr
DTh
Met
Val
Pro
DPTU
Trp
DPU
Phe
Lys
Ile
Leu

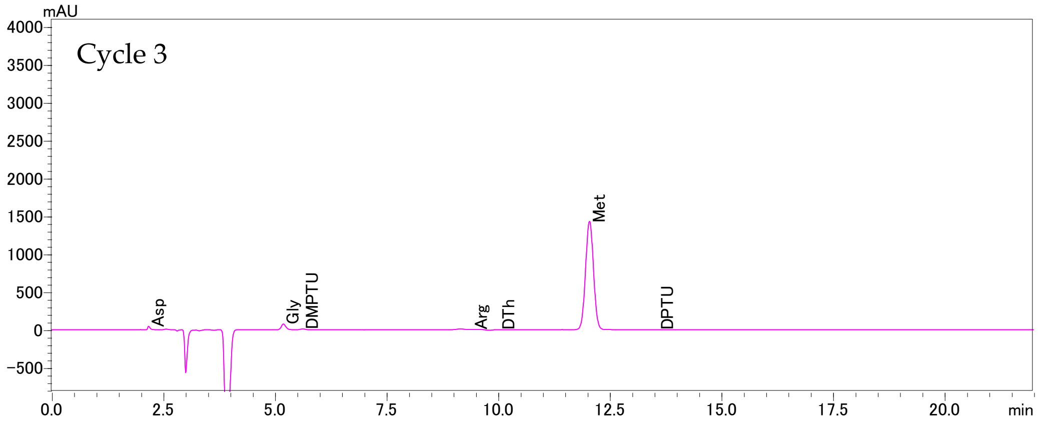
Cycle 3
mAU
4000
3500
3000
2500
2000
1500
1000
500
0
−500
0.0
2.5
5.0
7.5
10.0
12.5
15.0
17.5
20.0
min
Asp
Gly
DMPTU
Arg
DTh
Met
DPTU

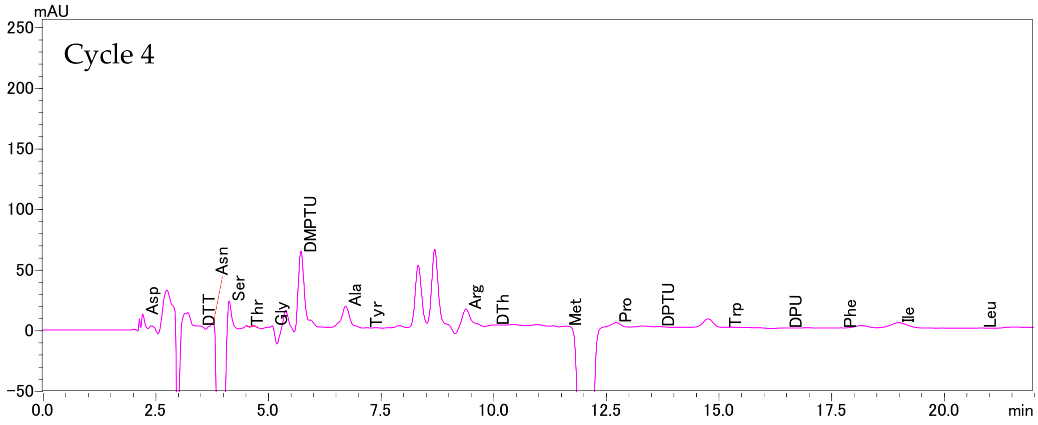
Cycle 4
mAU
250
200
150
100
50
0
−50
0.0
2.5
5.0
7.5
10.0
12.5
15.0
17.5
20.0
min
Asp
DTT
Asn
Ser
Thr
Gly
DMPTU
Ala
Tyr
Arg
DTh
Met
Pro
DPTU
Trp
DPU
Phe
Ile
Leu

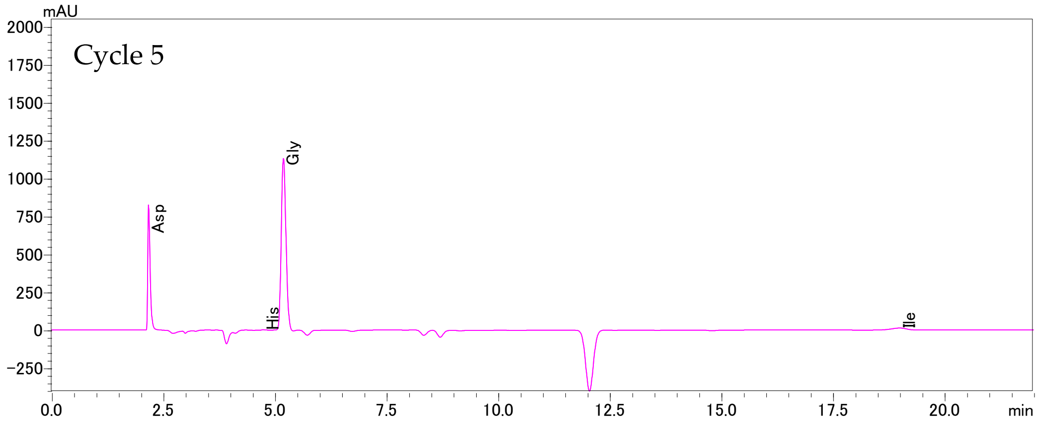
Cycle 5
mAU
2000
1750
1500
1250
1000
750
500
250
0
−250
0.0
2.5
5.0
7.5
10.0
12.5
15.0
17.5
20.0
min
Asp
His
Gly
Ile

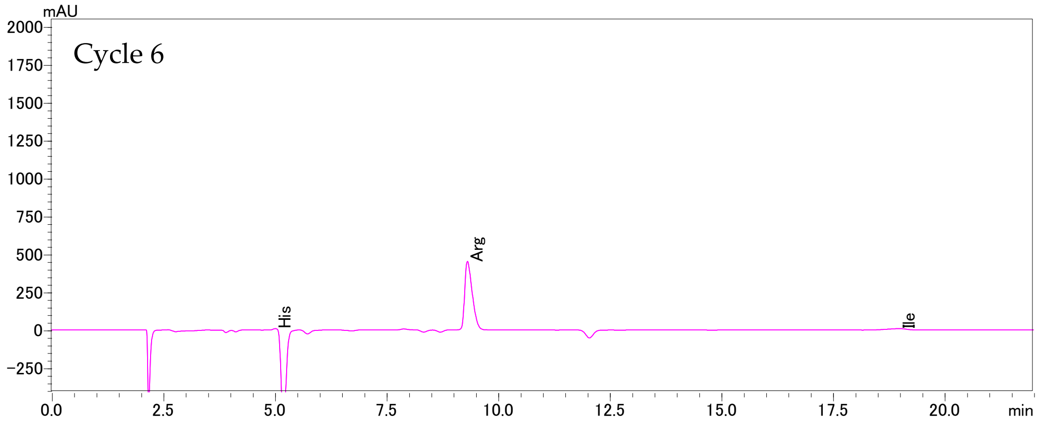

mAU
Cycle 6
2000
1750
1500
1250
1000
750
500
250
0
−250
His
Arg
Ile
0.0
2.5
5.0
7.5
10.0
12.5
15.0
17.5
20.0
min

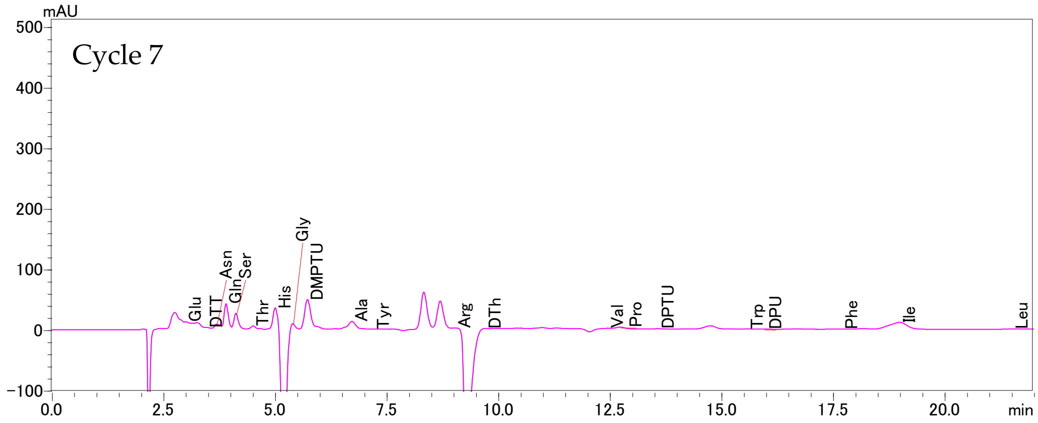

mAU
Cycle 7
500
400
300
200
100
0
−100
Glu
DTT
Asn
Gln
Ser
Thr
His
Gly
DMPTU
Ala
Tyr
Arg
DTh
Val
Pro
DPTU
Trp
DPU
Phe
Ile
Leu
0.0
2.5
5.0
7.5
10.0
12.5
15.0
17.5
20.0
min

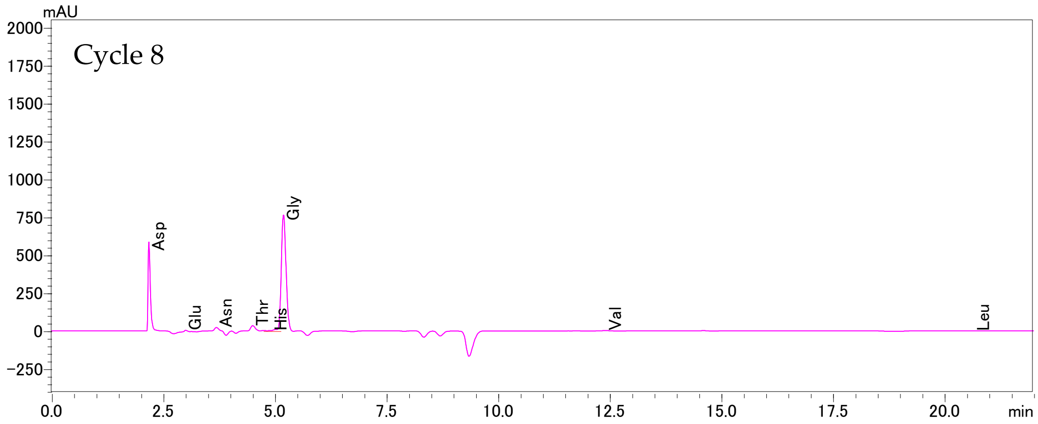

mAU
Cycle 8
2000
1750
1500
1250
1000
750
500
250
0
−250
Asp
Glu
Asn
Thr
His
Gly
Val
Leu
0.0
2.5
5.0
7.5
10.0
12.5
15.0
17.5
20.0
min

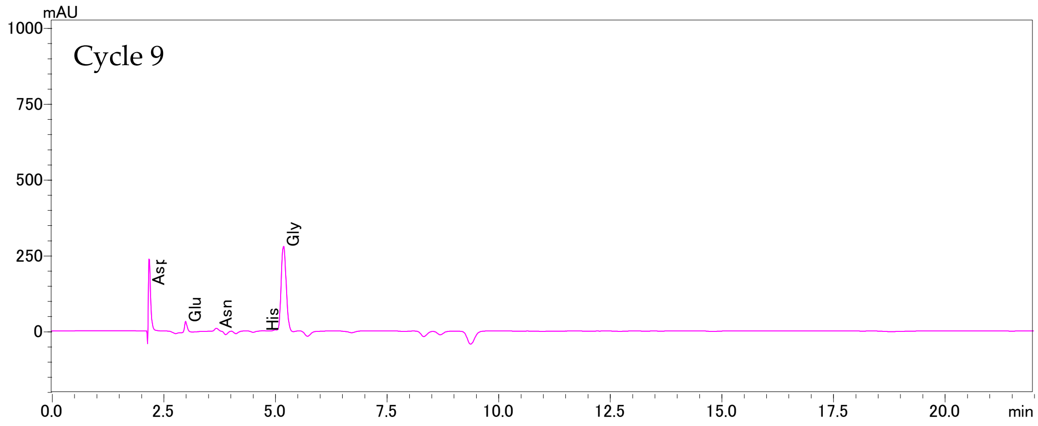

mAU
Cycle 9
1000
750
500
250
0
Glu
Asn
His
Gly
0.0
2.5
5.0
7.5
10.0
12.5
15.0
17.5
20.0
min

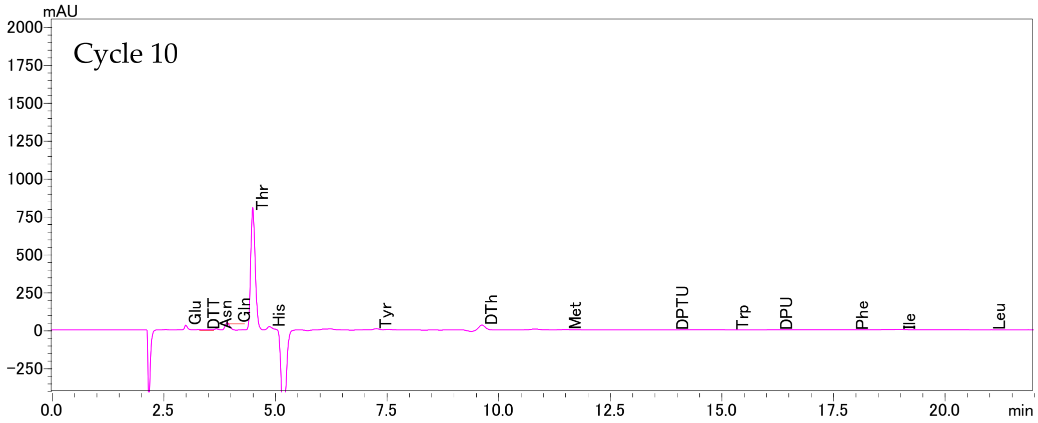

Cycle 10
mAU
2000
1750
1500
1250
1000
750
500
250
0
−250
Glu
DTT
Asn
Gln
Thr
His
Tyr
DTh
Met
DPTU
Trp
DPU
Phe
Ile
Leu
0.0
2.5
5.0
7.5
10.0
12.5
15.0
17.5
20.0
min

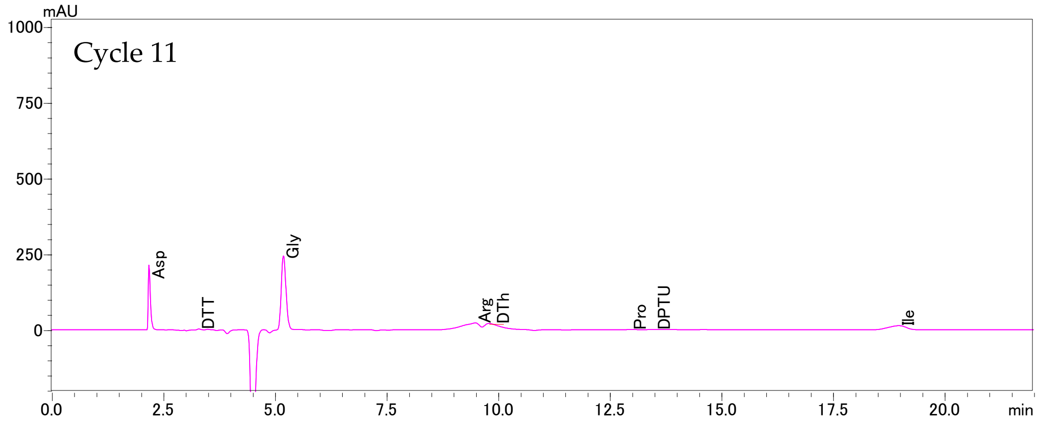

Cycle 11
mAU
1000
750
500
250
0
Asp
DTT
Gly
Arg
DTh
Pro
DPTU
Ile
0.0
2.5
5.0
7.5
10.0
12.5
15.0
17.5
20.0
min

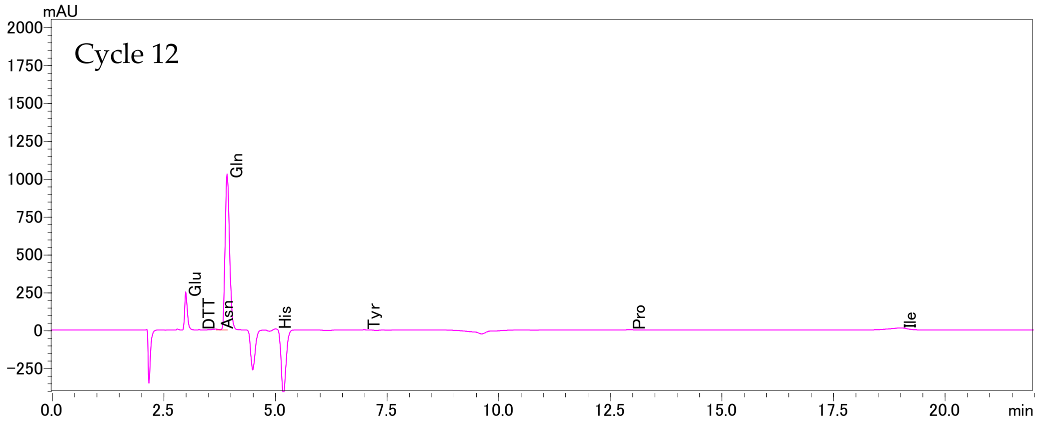

Cycle 12
mAU
2000
1750
1500
1250
1000
750
500
250
0
−250
Glu
DTT
Asn
Gln
His
Tyr
Pro
Ile
0.0
2.5
5.0
7.5
10.0
12.5
15.0
17.5
20.0
min

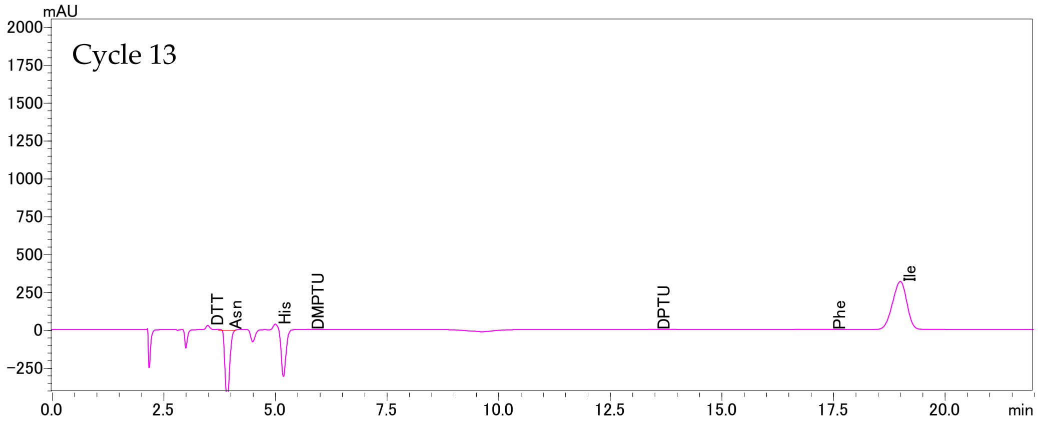

Cycle 13
mAU
2000
1750
1500
1250
1000
750
500
250
0
−250
DTT
Asn
His
DMPTU
DPTU
Phe
Ile
0.0
2.5
5.0
7.5
10.0
12.5
15.0
17.5
20.0
min

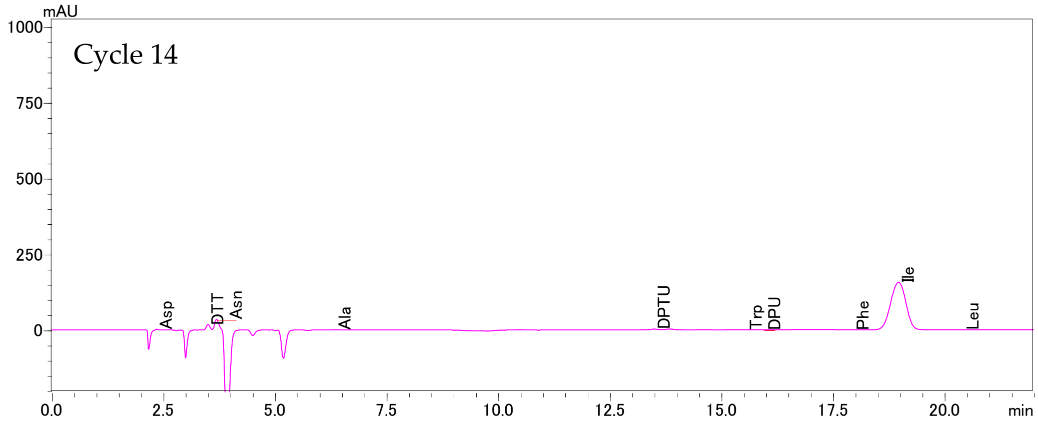
mAU
Cycle 14
1000
750
500
250
0
Asp
DTT
Asn
Ala
DPTU
Trp
DPU
Phe
Ile
Leu
0.0
2.5
5.0
7.5
10.0
12.5
15.0
17.5
20.0
min

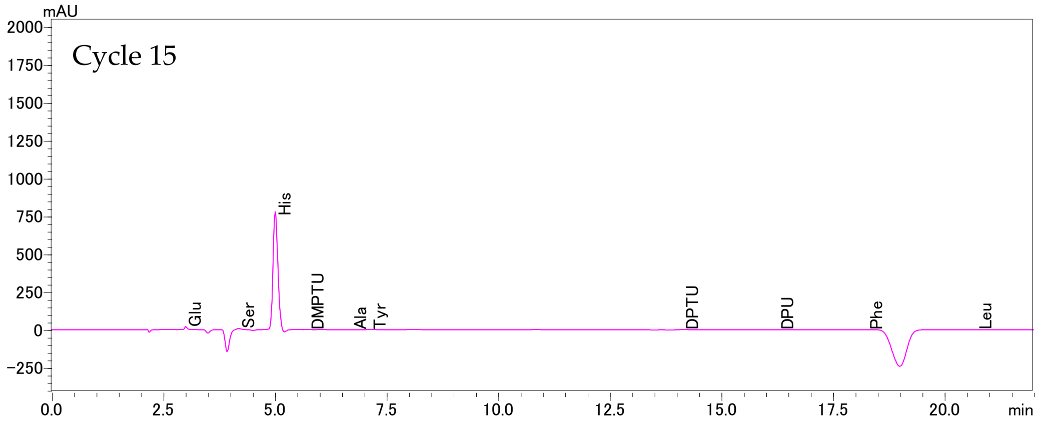
mAU
Cycle 15
2000
1750
1500
1250
1000
750
500
250
0
−250
Glu
Ser
His
DMPTU
Ala
Tyr
DPTU
DPU
Phe
Leu
0.0
2.5
5.0
7.5
10.0
12.5
15.0
17.5
20.0
min

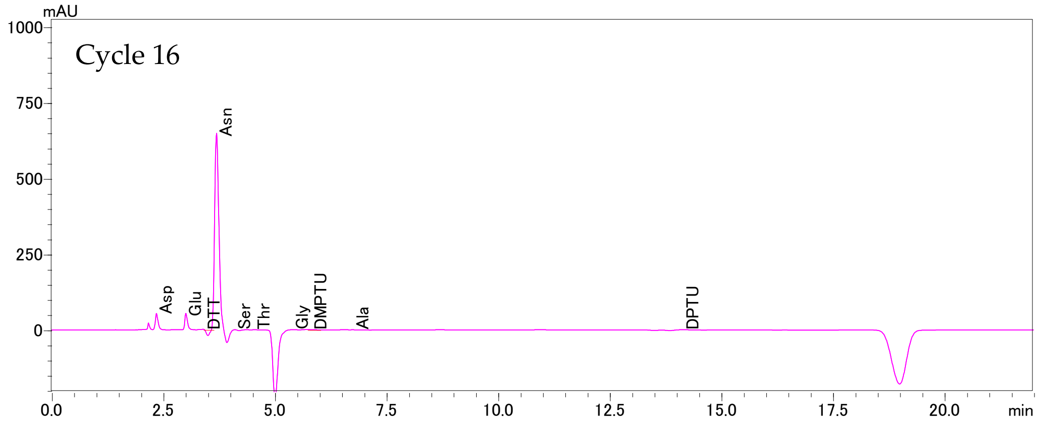
mAU
Cycle 16
1000
750
500
250
0
Asp
Glu
DTT
Asn
Ser
Thr
Gly
DMPTU
Ala
DPTU
0.0
2.5
5.0
7.5
10.0
12.5
15.0
17.5
20.0
min

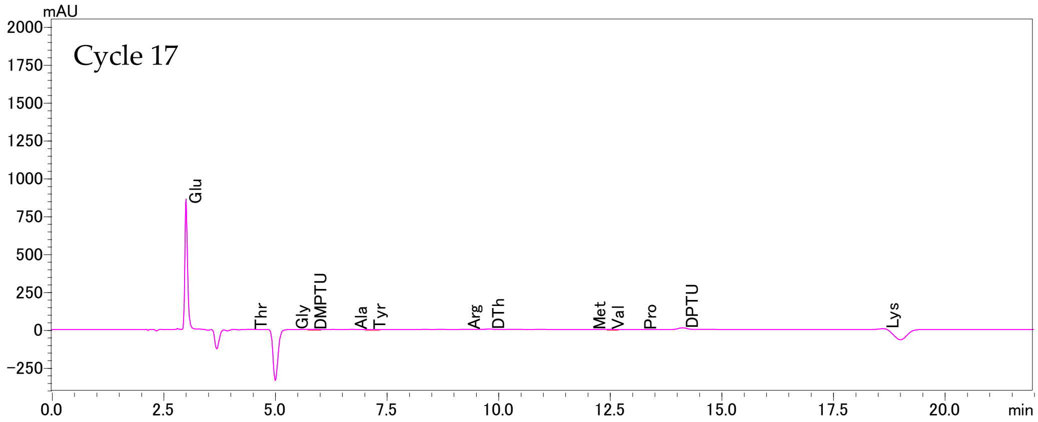
mAU
Cycle 17
2000
1750
1500
1250
1000
750
500
250
0
−250
Glu
Thr
Gly
DMPTU
Ala
Tyr
Arg
DTh
Met
Val
Pro
DPTU
Lys
0.0
2.5
5.0
7.5
10.0
12.5
15.0
17.5
20.0
min

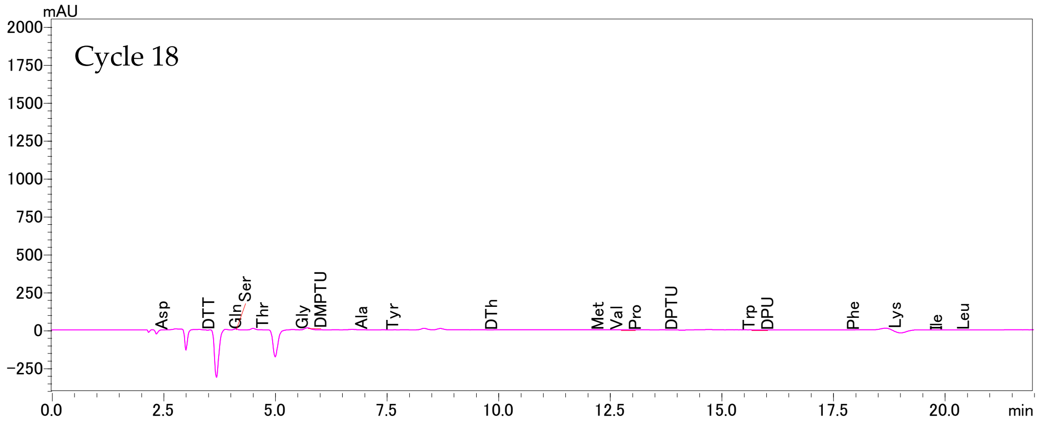
mAU
Cycle 18
Asp
DTT
Gln
Ser
Thr
Gly
DMPTU
Ala
Tyr
DTh
Met
Val
Pro
DPTU
Trp
DPU
Phe
Lys
Ile
Leu
min

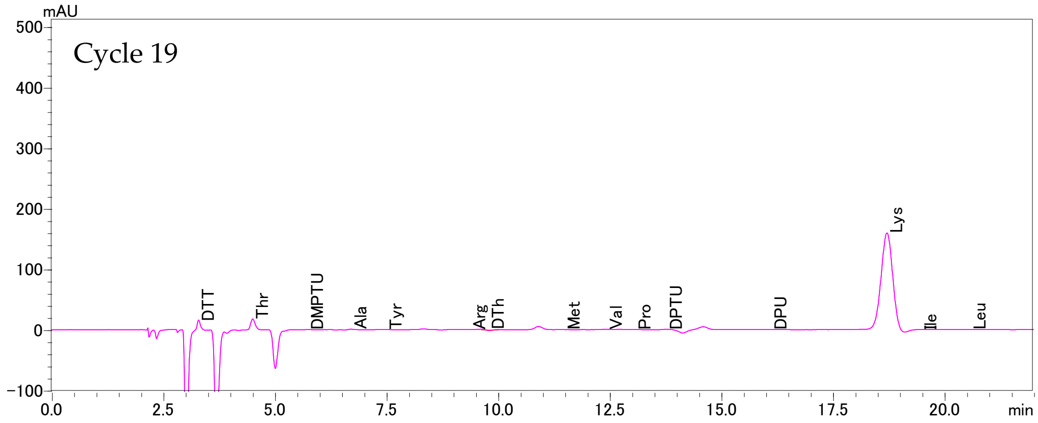
mAU
Cycle 19
DTT
Thr
DMPTU
Ala
Tyr
Arg
DTh
Met
Val
Pro
DPTU
DPU
Lys
Ile
Leu
min

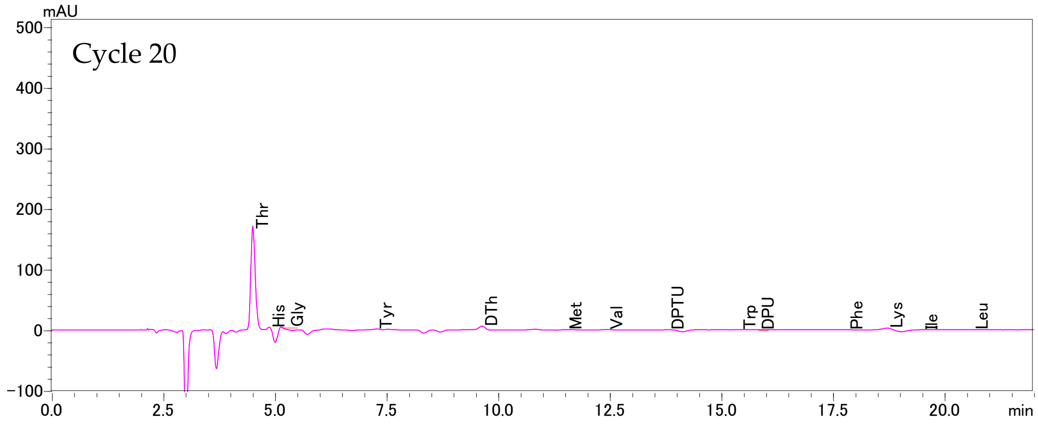
mAU
Cycle 20
Thr
His
Gly
Tyr
DTh
Met
Val
DPTU
Trp
DPU
Phe
Lys
Ile
Leu
min

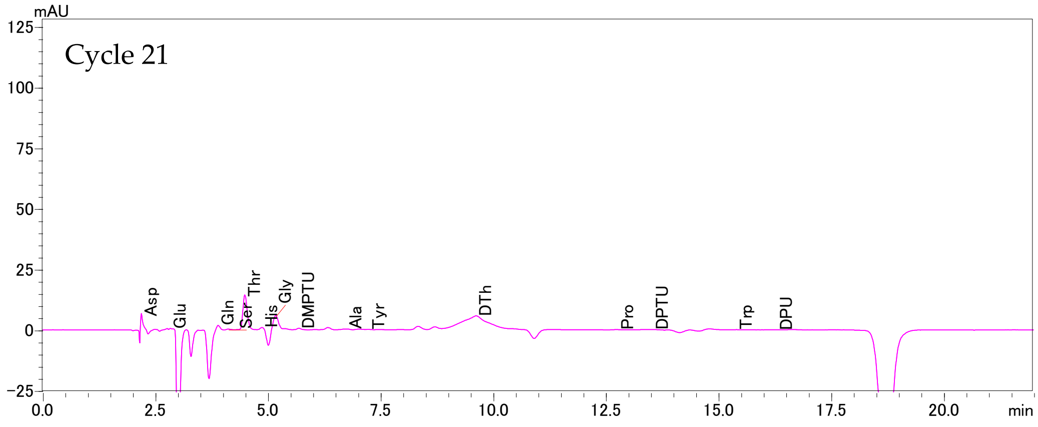
mAU
Cycle 21
Asp
Glu
Gln
Ser
Thr
His
Gly
DMPTU
Ala
Tyr
DTh
Pro
DPTU
Trp
DPU
min

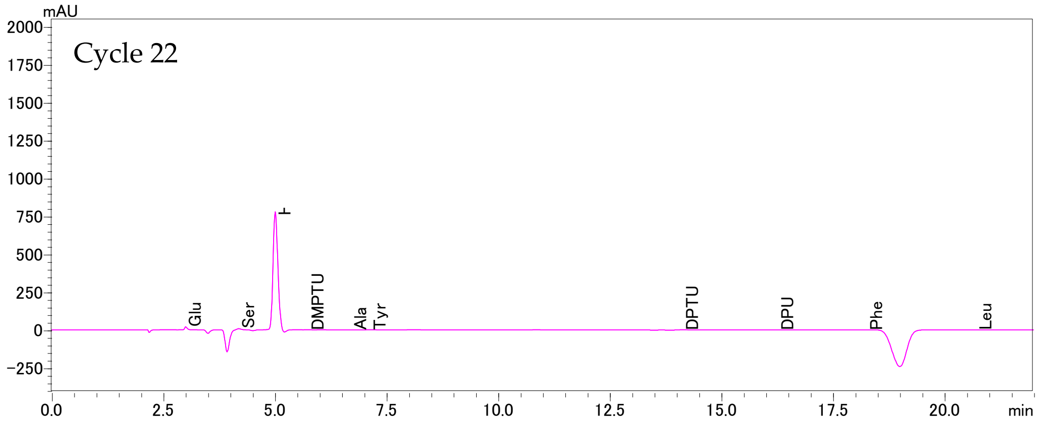
mAU
Cycle 22
Glu
Ser
DMPTU
Ala
Tyr
DPTU
DPU
Phe
Leu
min

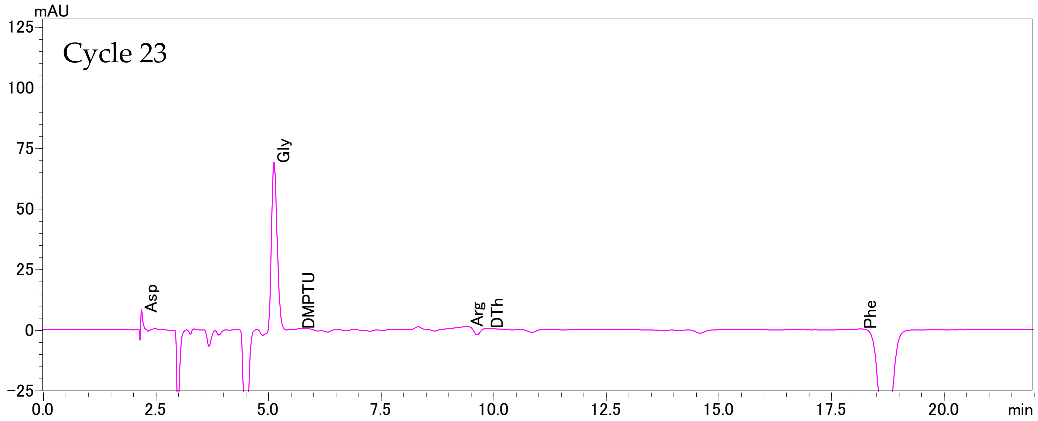
mAU
Cycle 23
Gly
Asp
DMPTU
Arg
DTh
Phe
min

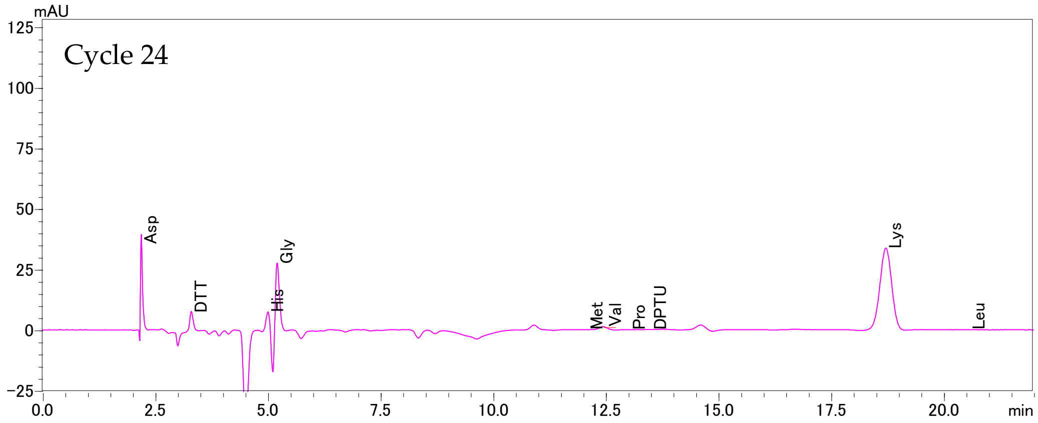
mAU
Cycle 24
Asp
DTT
Gly
His
Met
Val
Pro
DPTU
Lys
Leu
min

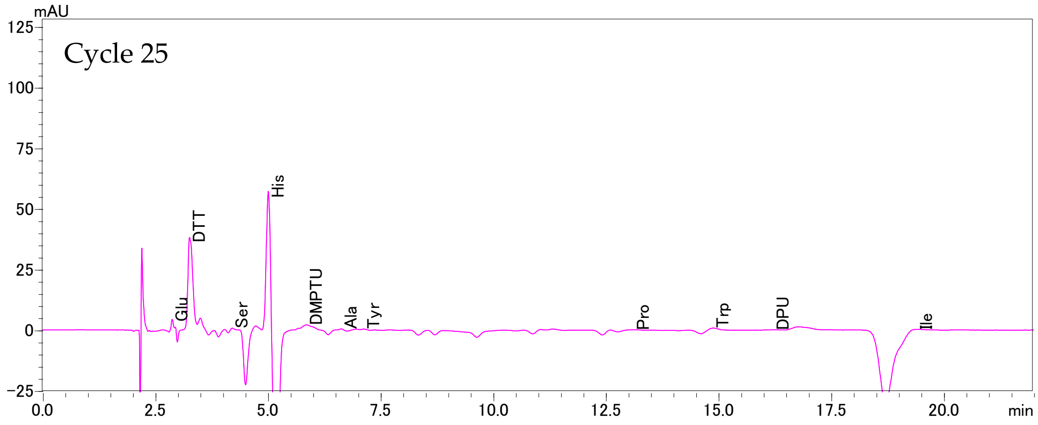
mAU
Cycle 25
His
DTT
Glu
Ser
DMPTU
Ala
Tyr
Pro
Trp
DPU
Ile
min

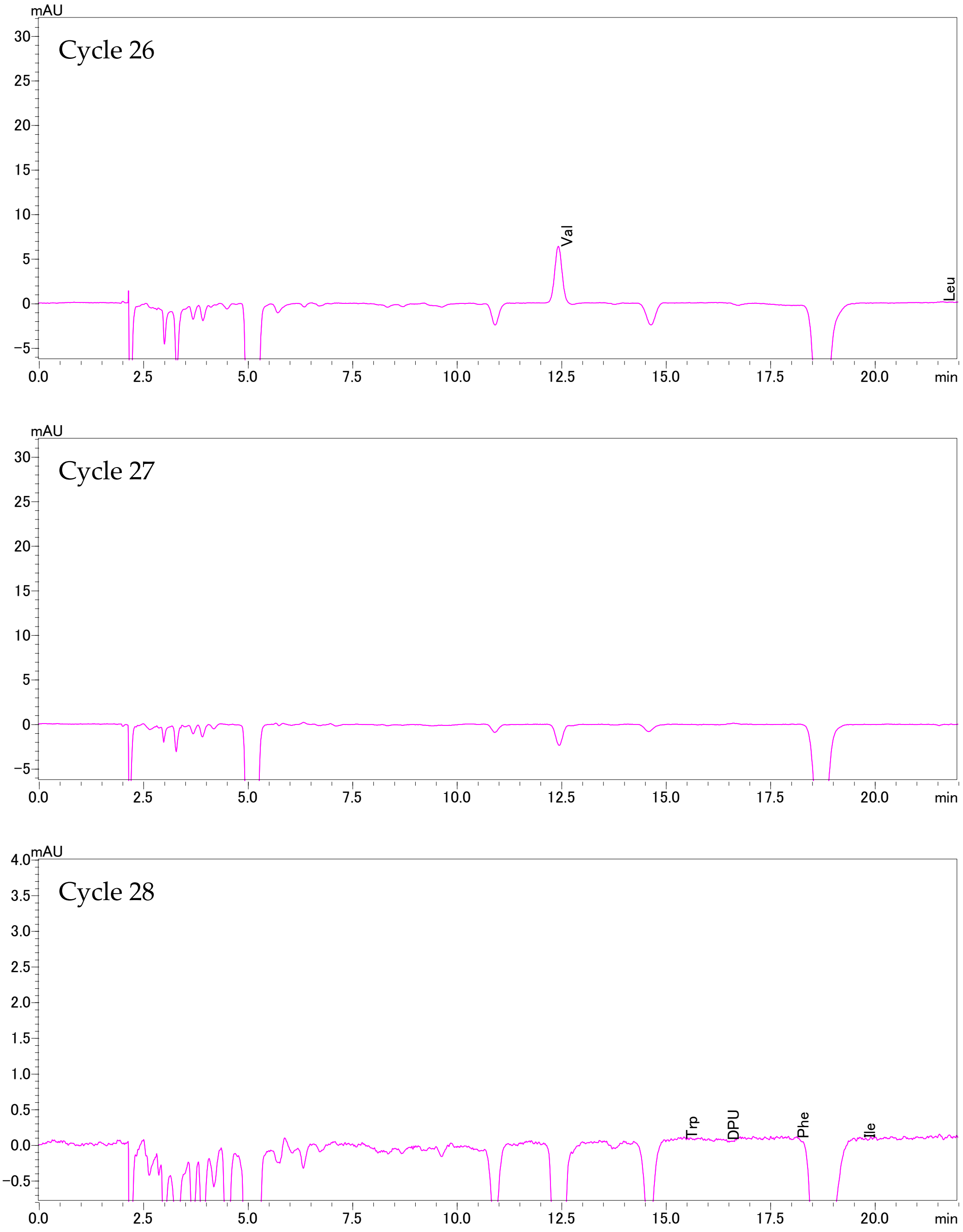

Figure S2. HPLC chromatograms of the amino acids released from each Edman degradation cycle of Vi14b.

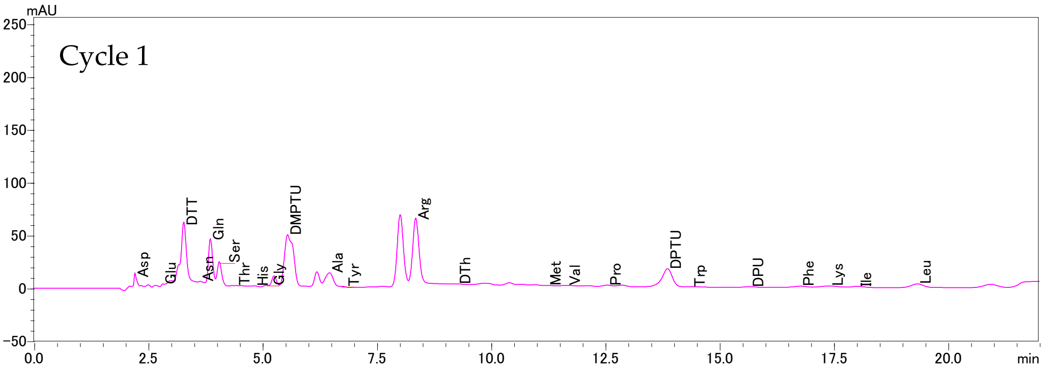

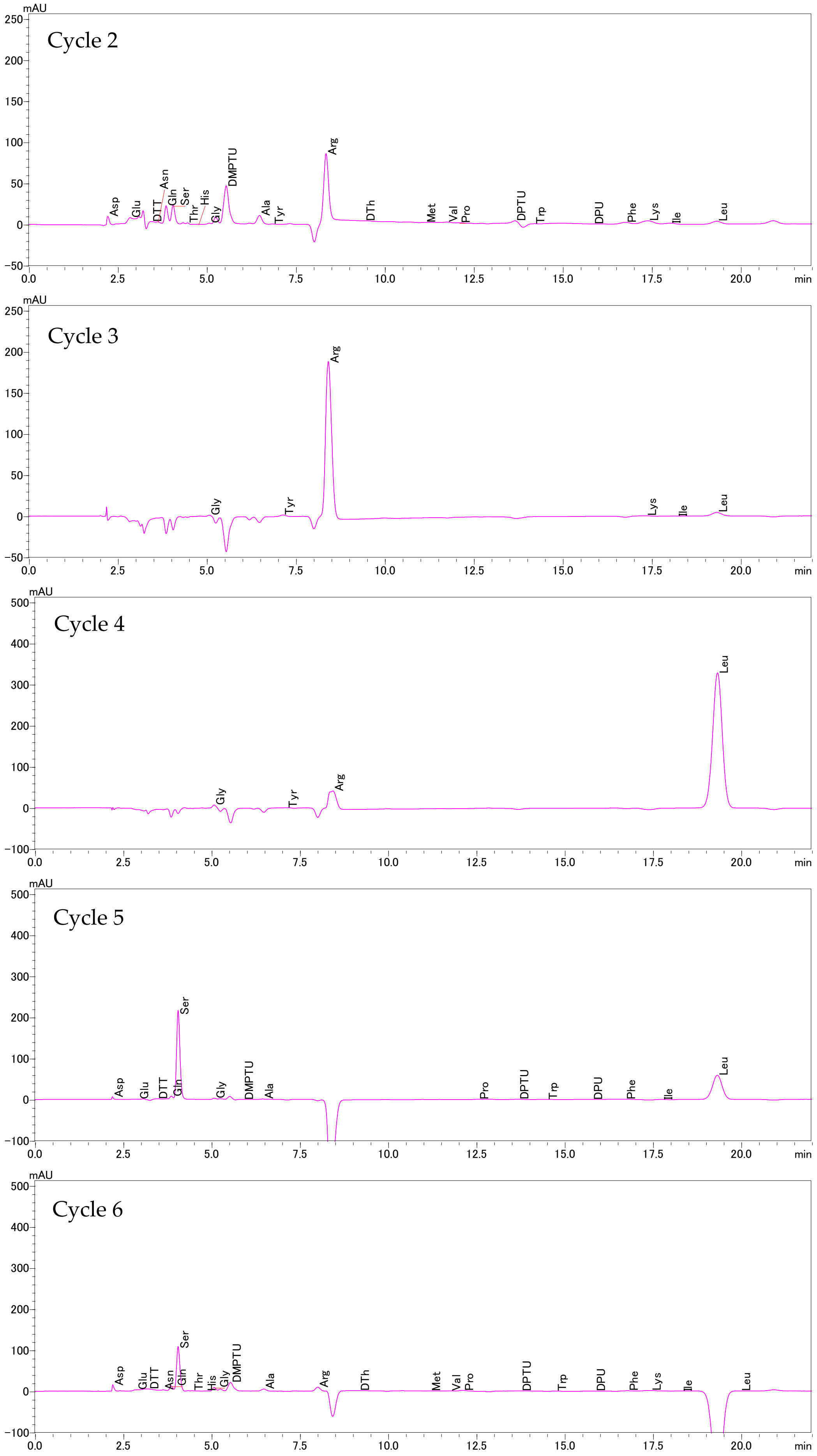
Cycle 2
mAU
min
Asp
Glu
DTT
Asn
Gln
Ser
Thr
His
Gly
DMPTU
Ala
Tyr
Arg
DTh
Met
Val
Pro
DPTU
Trp
DPU
Phe
Lys
Ile
Leu
Cycle 3
Gly
Tyr
Arg
Lys
Ile
Leu
Cycle 4
Gly
Tyr
Arg
Leu
Cycle 5
Asp
Glu
DTT
Gln
Ser
Gly
DMPTU
Ala
Pro
DPTU
Trp
DPU
Phe
Ile
Leu
Cycle 6
Asp
Glu
DTT
Asn
Gln
Ser
Thr
His
Gly
DMPTU
Ala
Arg
DTh
Met
Val
Pro
DPTU
Trp
DPU
Phe
Lys
Ile
Leu

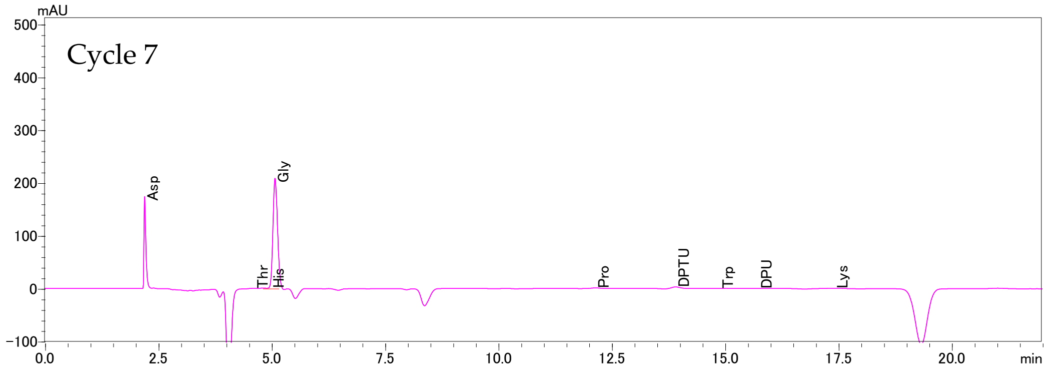

Cycle 7
mAU
500
400
300
200
100
0
−100
Asp
Gly
Thr
His
Pro
DPTU
Trp
DPU
Lys
0.0
2.5
5.0
7.5
10.0
12.5
15.0
17.5
20.0
min

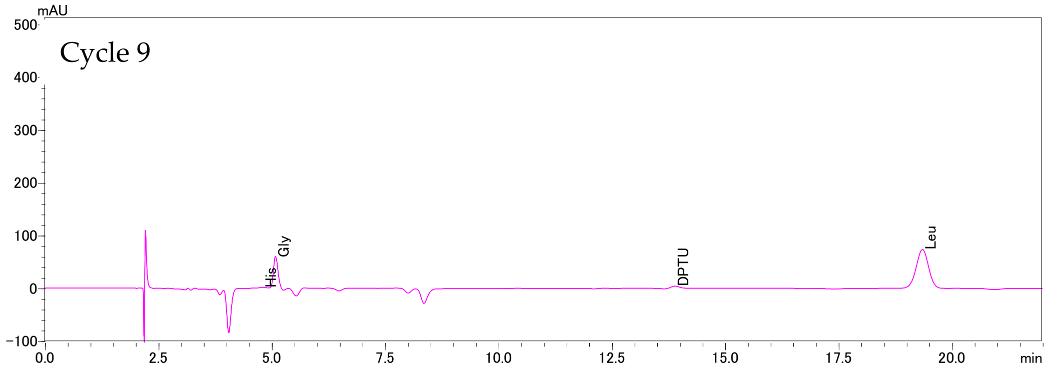

Cycle 9
mAU
500
400
300
200
100
0
−100
His
Gly
DPTU
Leu
0.0
2.5
5.0
7.5
10.0
12.5
15.0
17.5
20.0
min

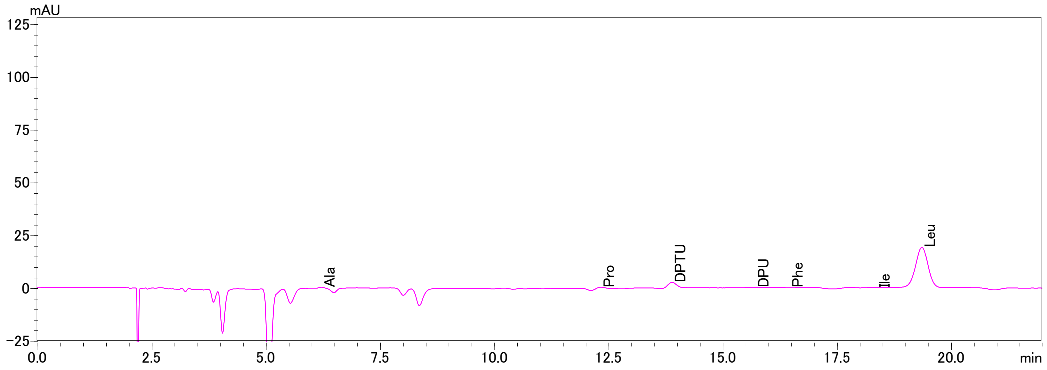

mAU
125
100
75
50
25
0
−25
Ala
Pro
DPTU
DPU
Phe
Ile
Leu
0.0
2.5
5.0
7.5
10.0
12.5
15.0
17.5
20.0
min

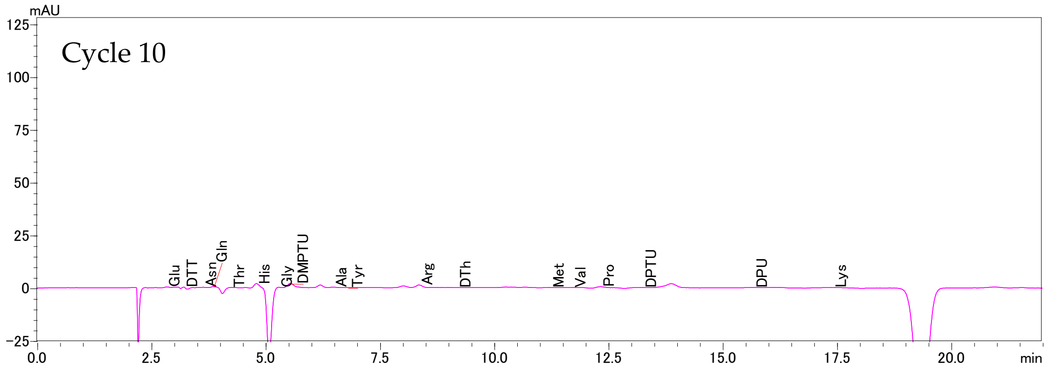

Cycle 10
mAU
125
100
75
50
25
0
−25
Glu
DTT
Asn
Gln
Thr
His
Gly
DMPTU
Ala
Tyr
Arg
DTh
Met
Val
Pro
DPTU
DPU
Lys
0.0
2.5
5.0
7.5
10.0
12.5
15.0
17.5
20.0
min

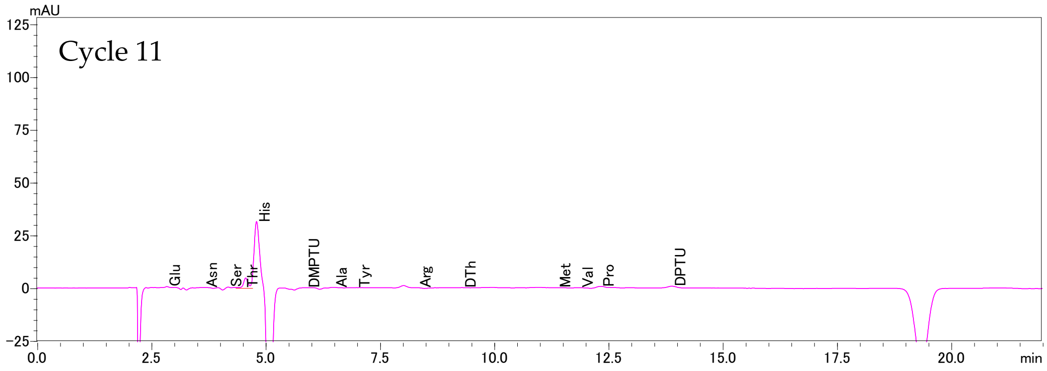

Cycle 11
mAU
125
100
75
50
25
0
−25
Glu
Asn
Ser
Thr
His
DMPTU
Ala
Tyr
Arg
DTh
Met
Val
Pro
DPTU
0.0
2.5
5.0
7.5
10.0
12.5
15.0
17.5
20.0
min

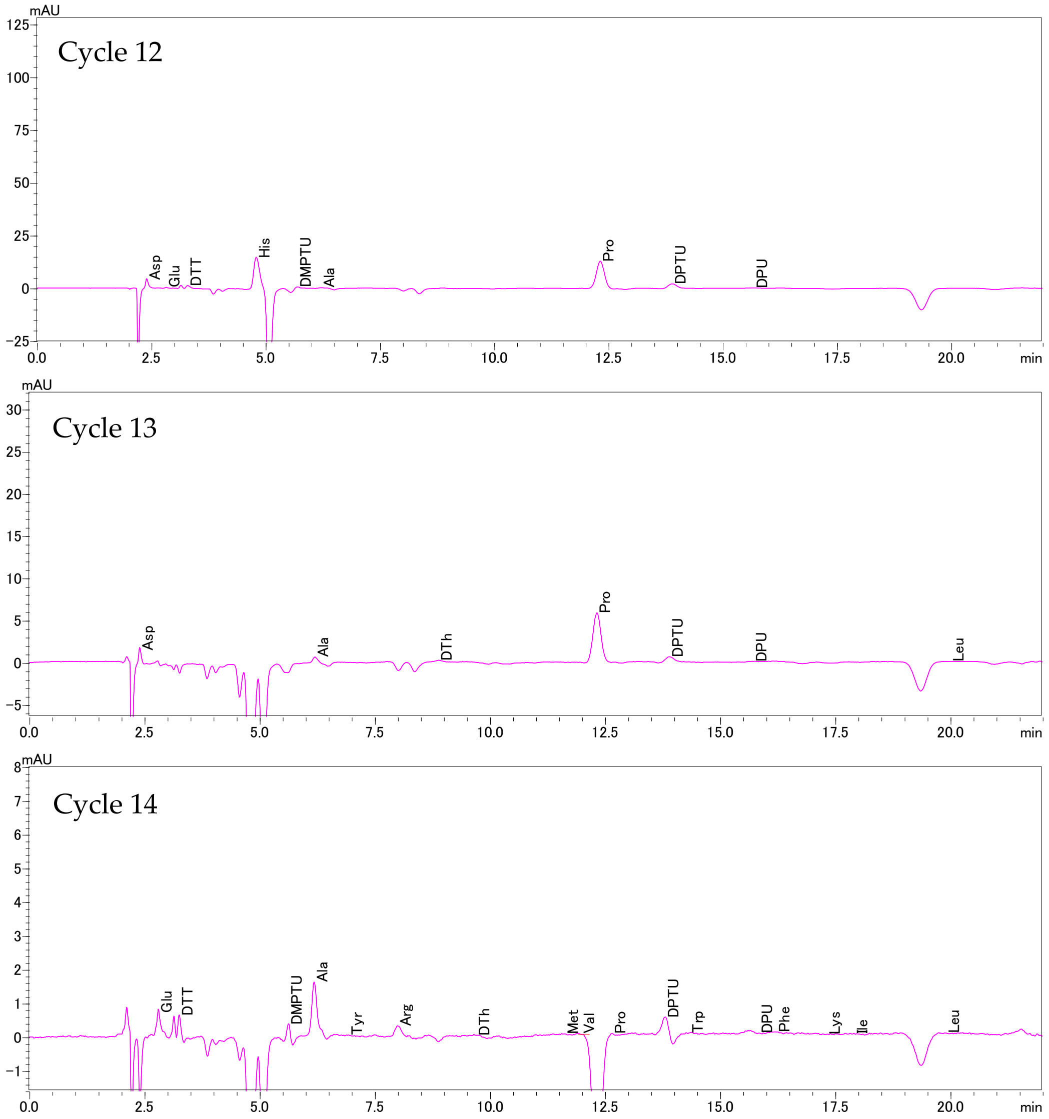

Figure S3. HPLC chromatograms of the amino acids released from each Edman degradation cycle of Mr3d.1.

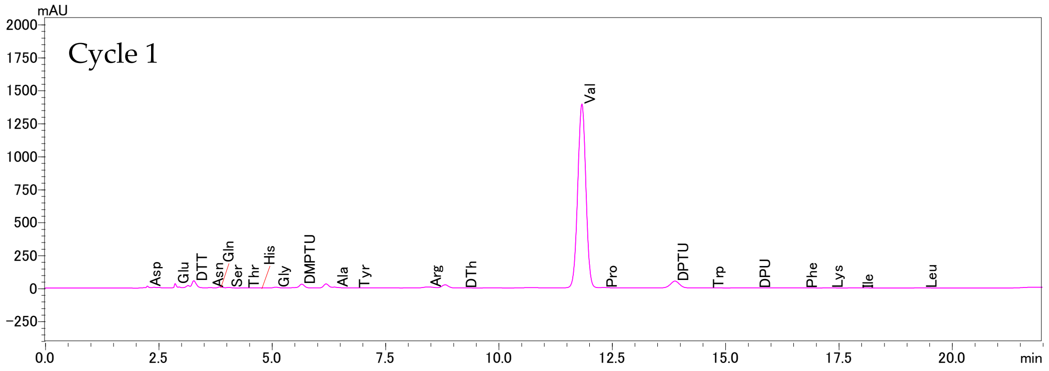

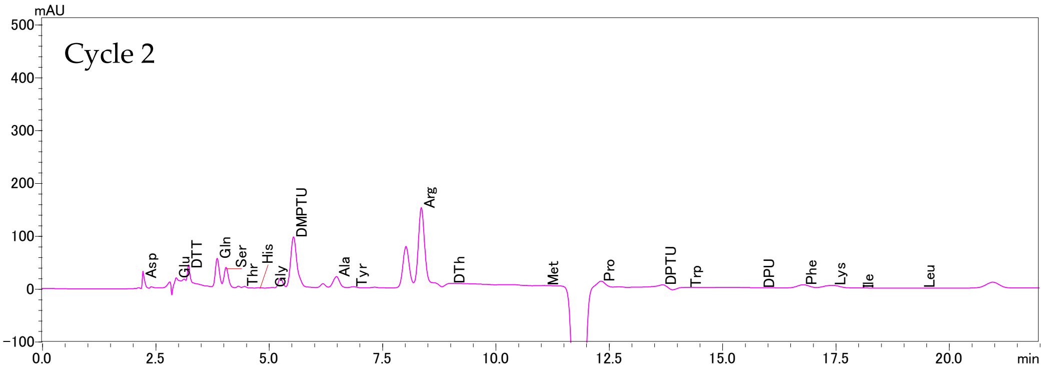
Cycle 2
mAU
500
400
300
200
100
0
−100
0.0
2.5
5.0
7.5
10.0
12.5
15.0
17.5
20.0
min
Asp
Glu
DTT
Gln
Ser
Thr
His
Gly
DMPTU
Ala
Tyr
Arg
DTh
Met
Pro
DPTU
Trp
DPU
Phe
Lys
Ile
Leu

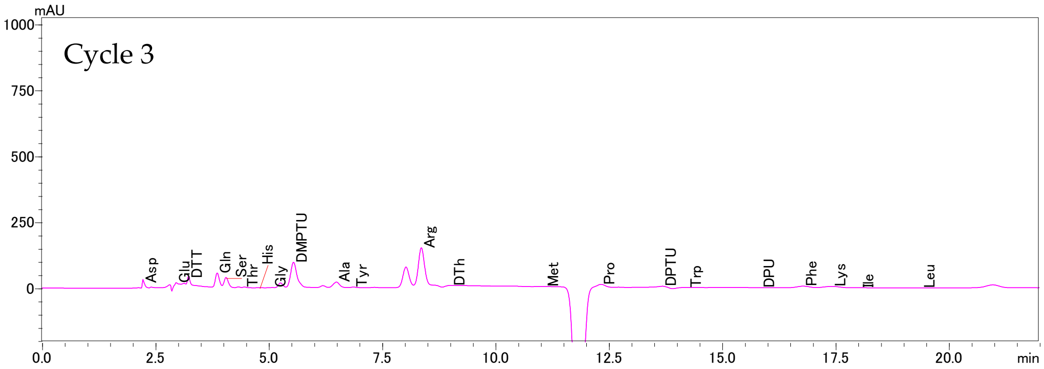
Cycle 3
mAU
1000
750
500
250
0
0.0
2.5
5.0
7.5
10.0
12.5
15.0
17.5
20.0
min
Asp
Glu
DTT
Gln
Ser
Thr
His
Gly
DMPTU
Ala
Tyr
Arg
DTh
Met
Pro
DPTU
Trp
DPU
Phe
Lys
Ile
Leu

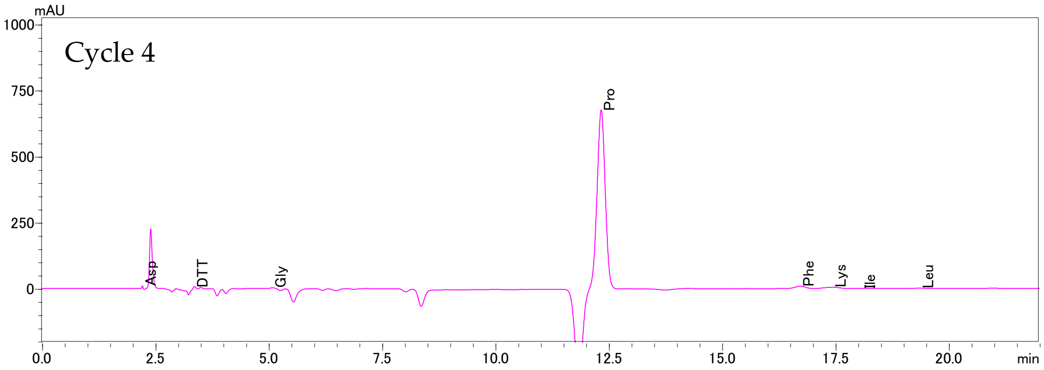
Cycle 4
mAU
1000
750
500
250
0
0.0
2.5
5.0
7.5
10.0
12.5
15.0
17.5
20.0
min
Asp
DTT
Gly
Pro
Phe
Lys
Ile
Leu

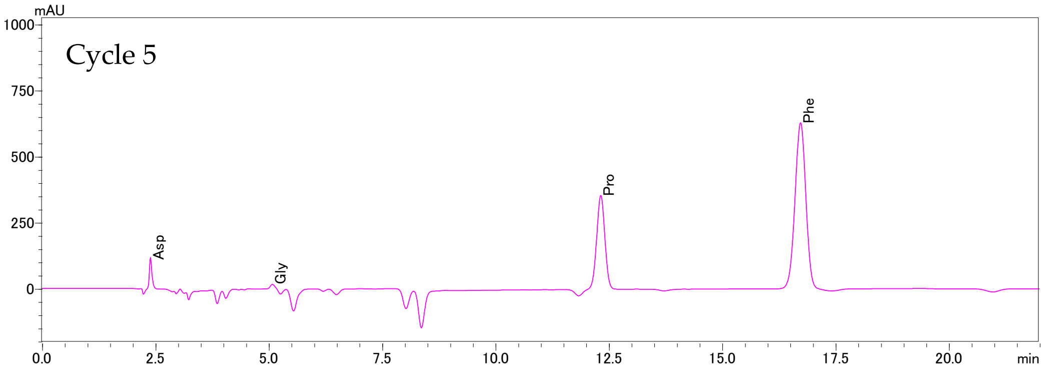
Cycle 5
mAU
1000
750
500
250
0
0.0
2.5
5.0
7.5
10.0
12.5
15.0
17.5
20.0
min
Asp
Gly
Pro
Phe

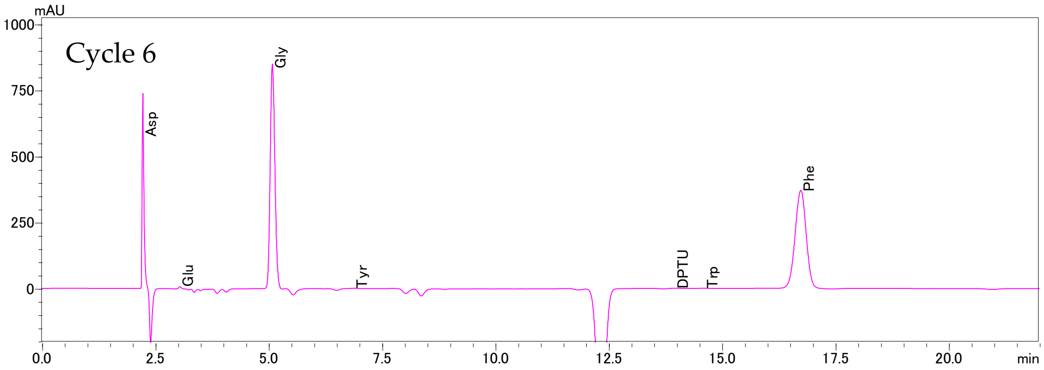
Cycle 6
mAU
1000
750
500
250
0
0.0
2.5
5.0
7.5
10.0
12.5
15.0
17.5
20.0
min
Asp
Glu
Gly
Tyr
DPTU
Trp
Phe

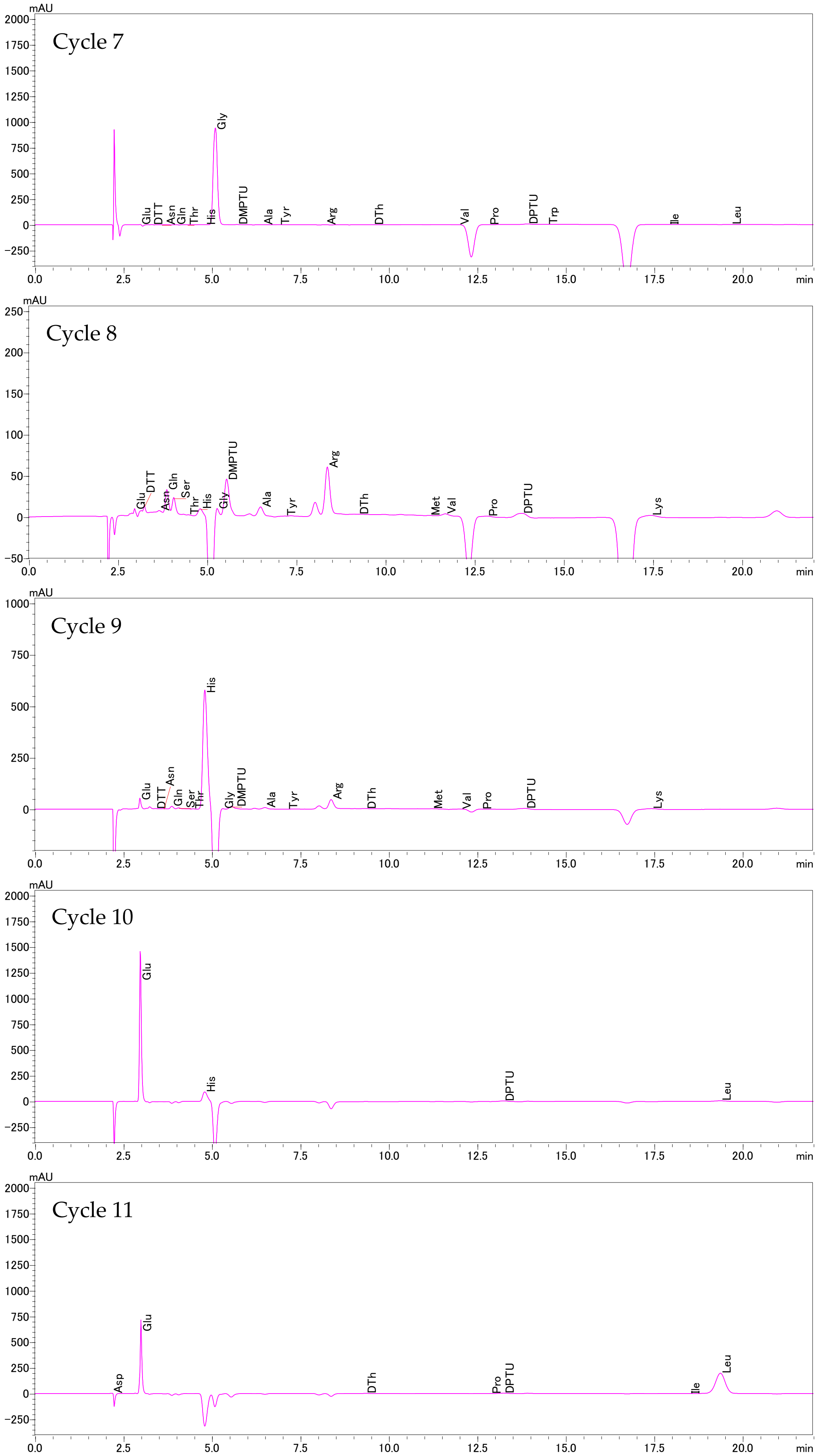
Cycle 7
mAU
min
Gly
Glu
DTT
Asn
Gln
Thr
His
DMPTU
Ala
Tyr
Arg
DTh
Val
Pro
DPTU
Trp
Ile
Leu
Cycle 8
Glu
DTT
Asn
Gln
Ser
Thr
His
Gly
DMPTU
Ala
Tyr
Arg
DTh
Met
Val
Pro
DPTU
Lys
Cycle 9
His
Glu
DTT
Asn
Gln
Ser
Thr
Gly
DMPTU
Ala
Tyr
Arg
DTh
Met
Val
Pro
DPTU
Lys
Cycle 10
Glu
His
DPTU
Leu
Cycle 11
Glu
Asp
DTh
Pro
DPTU
Ile
Leu

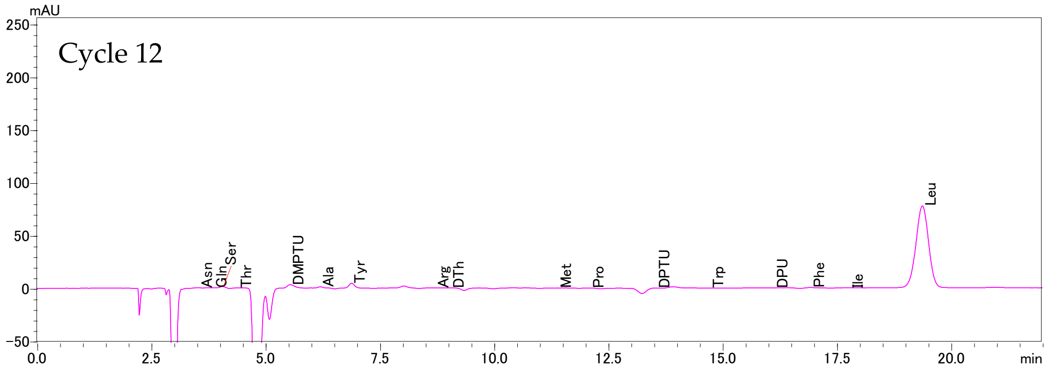
Cycle 12
mAU
250
200
150
100
50
0
−50
0.0
2.5
5.0
7.5
10.0
12.5
15.0
17.5
20.0
min
Asn
Gln
Ser
Thr
DMPTU
Ala
Tyr
Arg
DTh
Met
Pro
DPTU
Trp
DPU
Phe
Ile
Leu

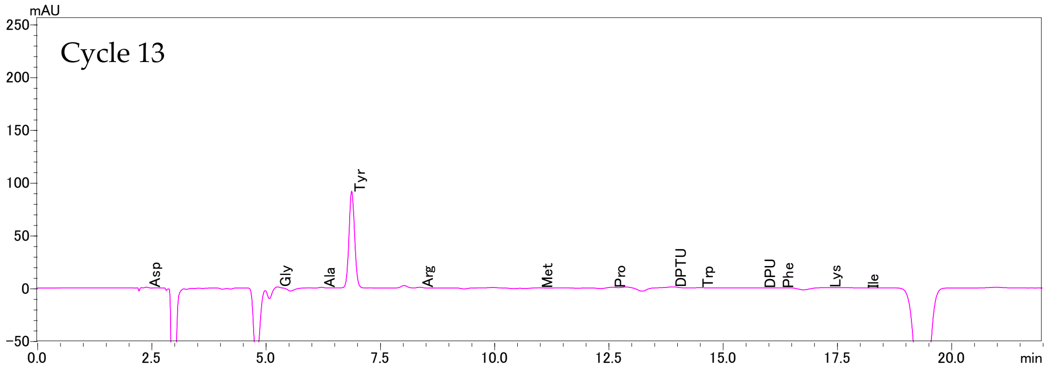
Cycle 13
mAU
250
200
150
100
50
0
−50
0.0
2.5
5.0
7.5
10.0
12.5
15.0
17.5
20.0
min
Asp
Gly
Ala
Tyr
Arg
Met
Pro
DPTU
Trp
DPU
Phe
Lys
Ile

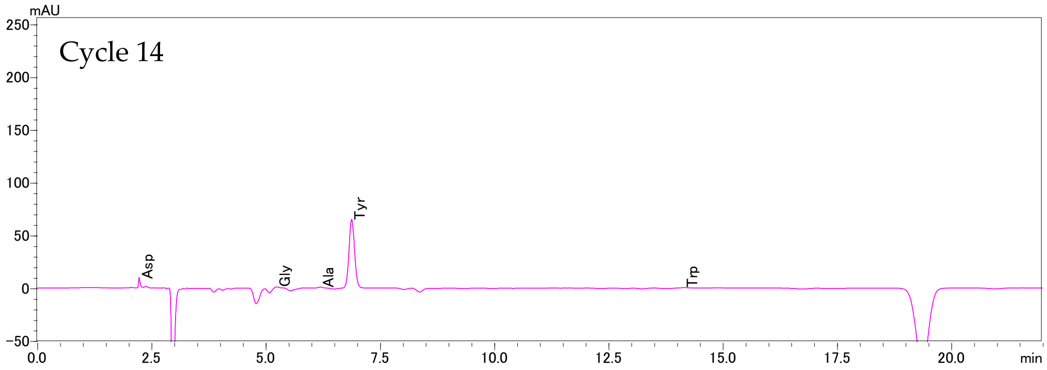
Cycle 14
mAU
250
200
150
100
50
0
−50
0.0
2.5
5.0
7.5
10.0
12.5
15.0
17.5
20.0
min
Asp
Gly
Ala
Tyr
Trp

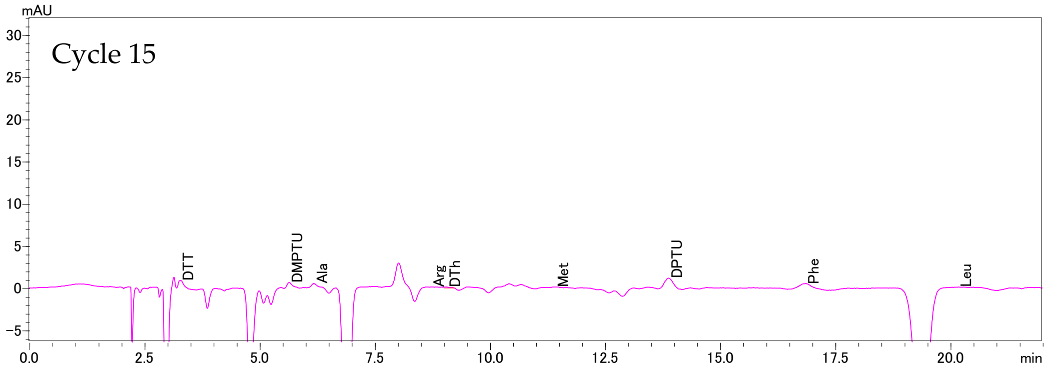
Cycle 15
mAU
30
25
20
15
10
5
0
−5
0.0
2.5
5.0
7.5
10.0
12.5
15.0
17.5
20.0
min
DTT
DMPTU
Ala
Arg
DTh
Met
DPTU
Phe
Leu

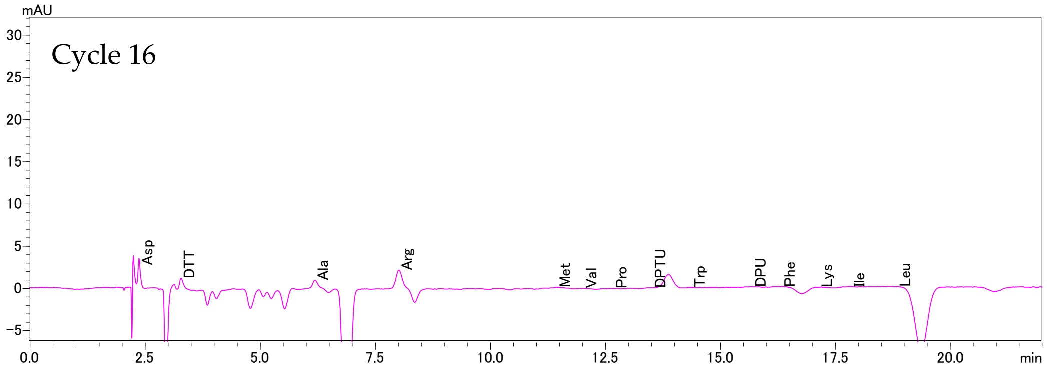
Cycle 16
mAU
30
25
20
15
10
5
0
−5
0.0
2.5
5.0
7.5
10.0
12.5
15.0
17.5
20.0
min
Asp
DTT
Ala
Arg
Met
Val
Pro
DPTU
Trp
DPU
Phe
Lys
Ile
Leu

Figure S4. HPLC chromatograms of the amino acids released from each Edman degradation cycle of Mr3e.1.

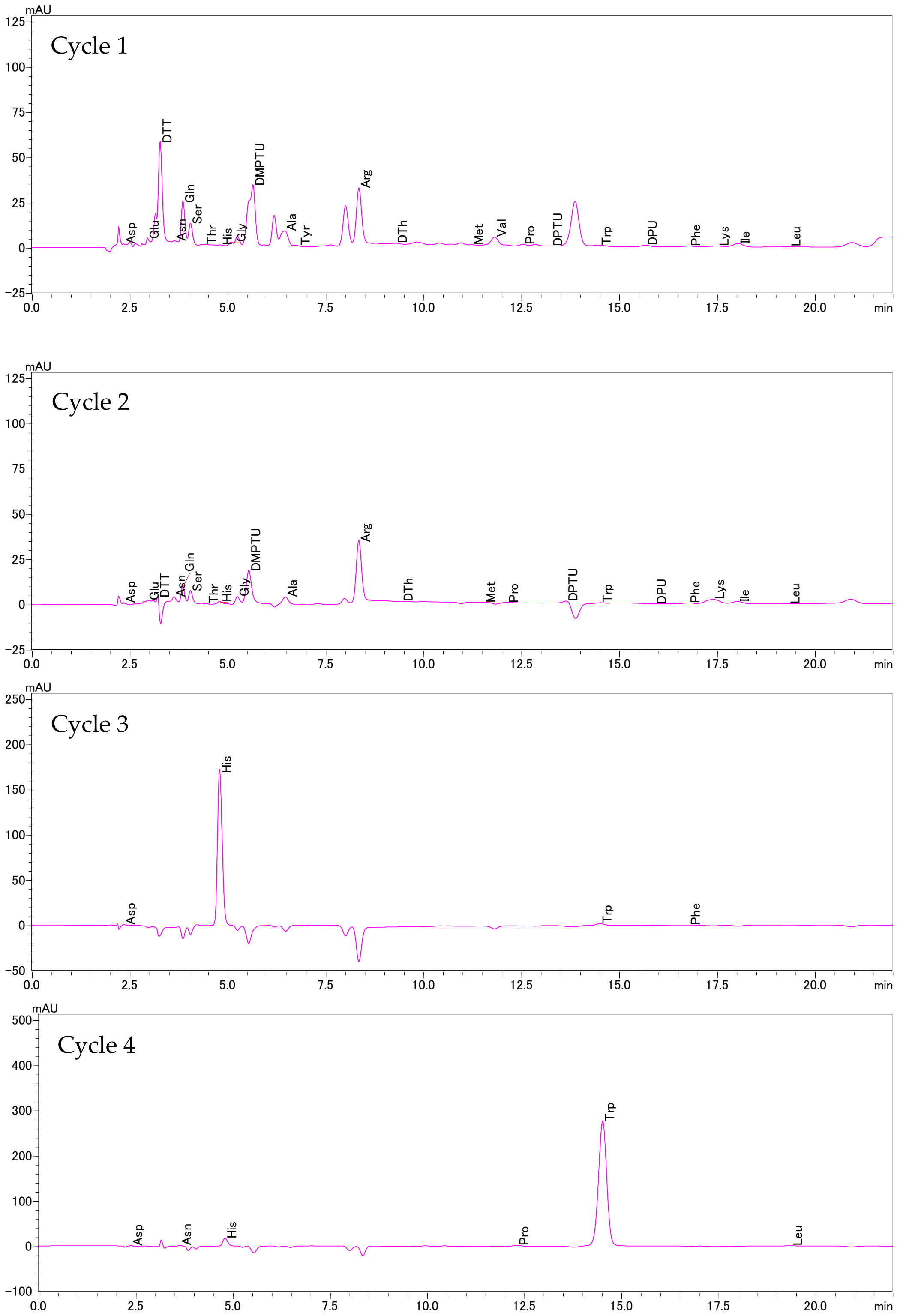

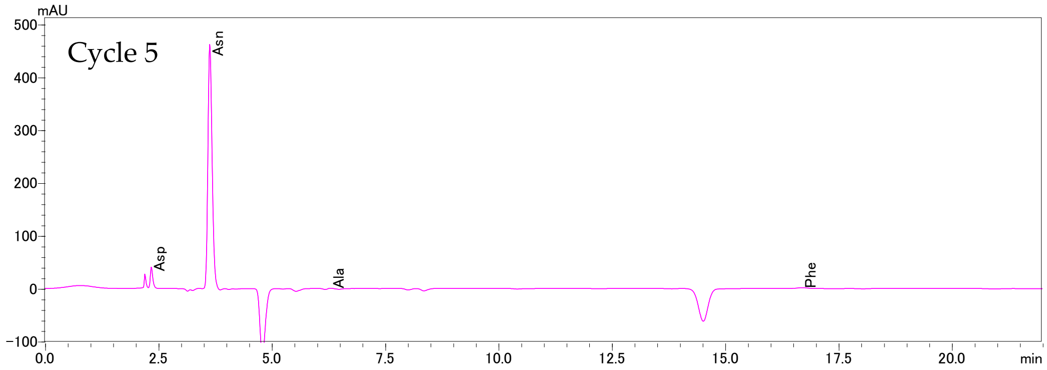
Cycle 5
mAU
500
400
300
200
100
0
−100
Asp
Asn
Ala
Phe
0.0
2.5
5.0
7.5
10.0
12.5
15.0
17.5
20.0
min

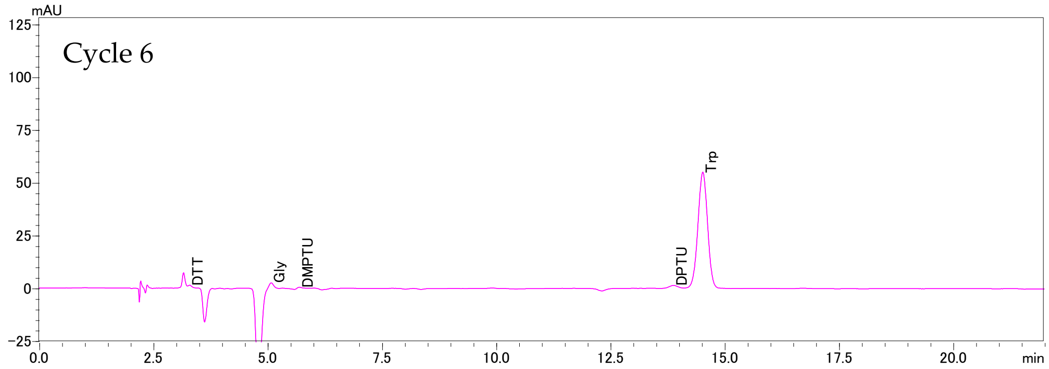
Cycle 6
mAU
125
100
75
50
25
0
−25
DTT
Gly
DMPTU
DPTU
Trp
0.0
2.5
5.0
7.5
10.0
12.5
15.0
17.5
20.0
min

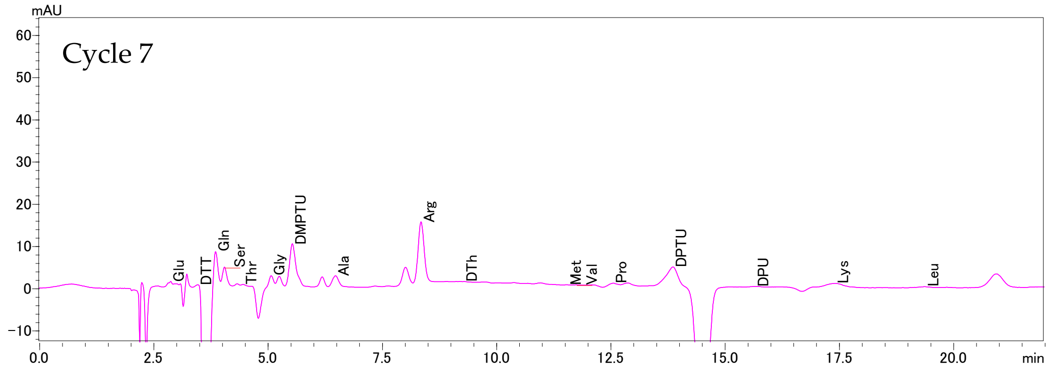
Cycle 7
mAU
60
50
40
30
20
10
0
−10
Glu
DTT
Gln
Ser
Thr
Gly
DMPTU
Ala
Arg
DTh
Met
Val
Pro
DPTU
DPU
Lys
Leu
0.0
2.5
5.0
7.5
10.0
12.5
15.0
17.5
20.0
min

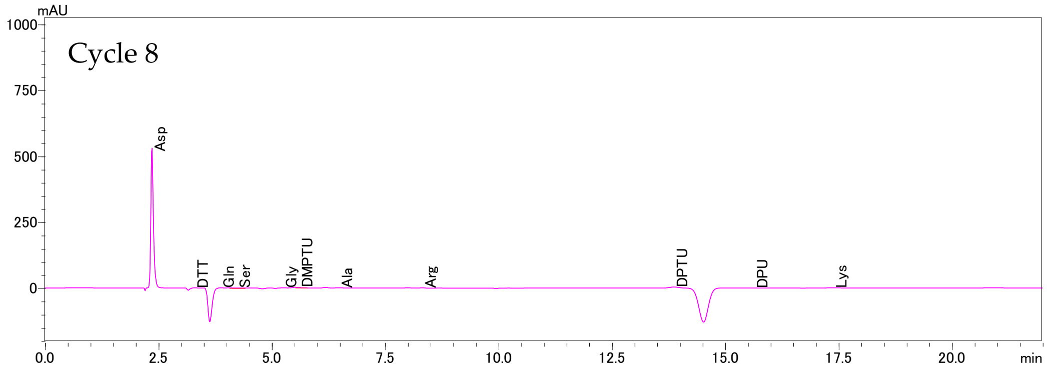
Cycle 8
mAU
1000
750
500
250
0
Asp
DTT
Gln
Ser
Gly
DMPTU
Ala
Arg
DPTU
DPU
Lys
0.0
2.5
5.0
7.5
10.0
12.5
15.0
17.5
20.0
min

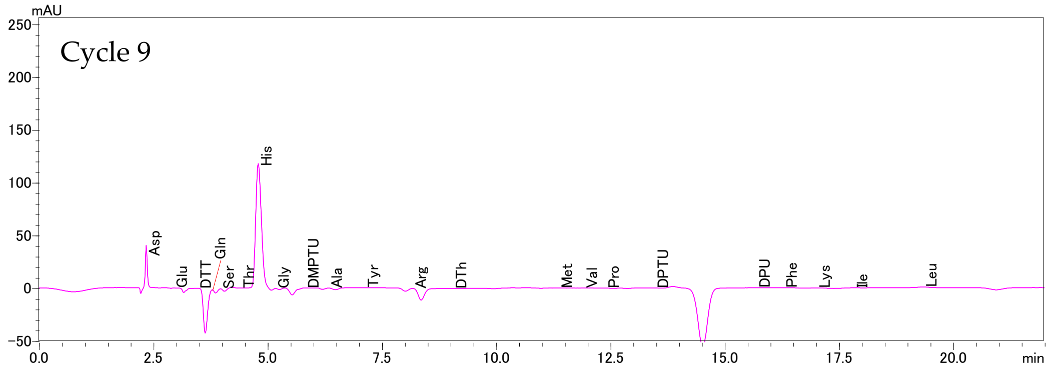
Cycle 9
mAU
250
200
150
100
50
0
−50
Asp
Glu
DTT
Gln
Ser
Thr
His
Gly
DMPTU
Ala
Tyr
Arg
DTh
Met
Val
Pro
DPTU
DPU
Phe
Lys
Ile
Leu
0.0
2.5
5.0
7.5
10.0
12.5
15.0
17.5
20.0
min

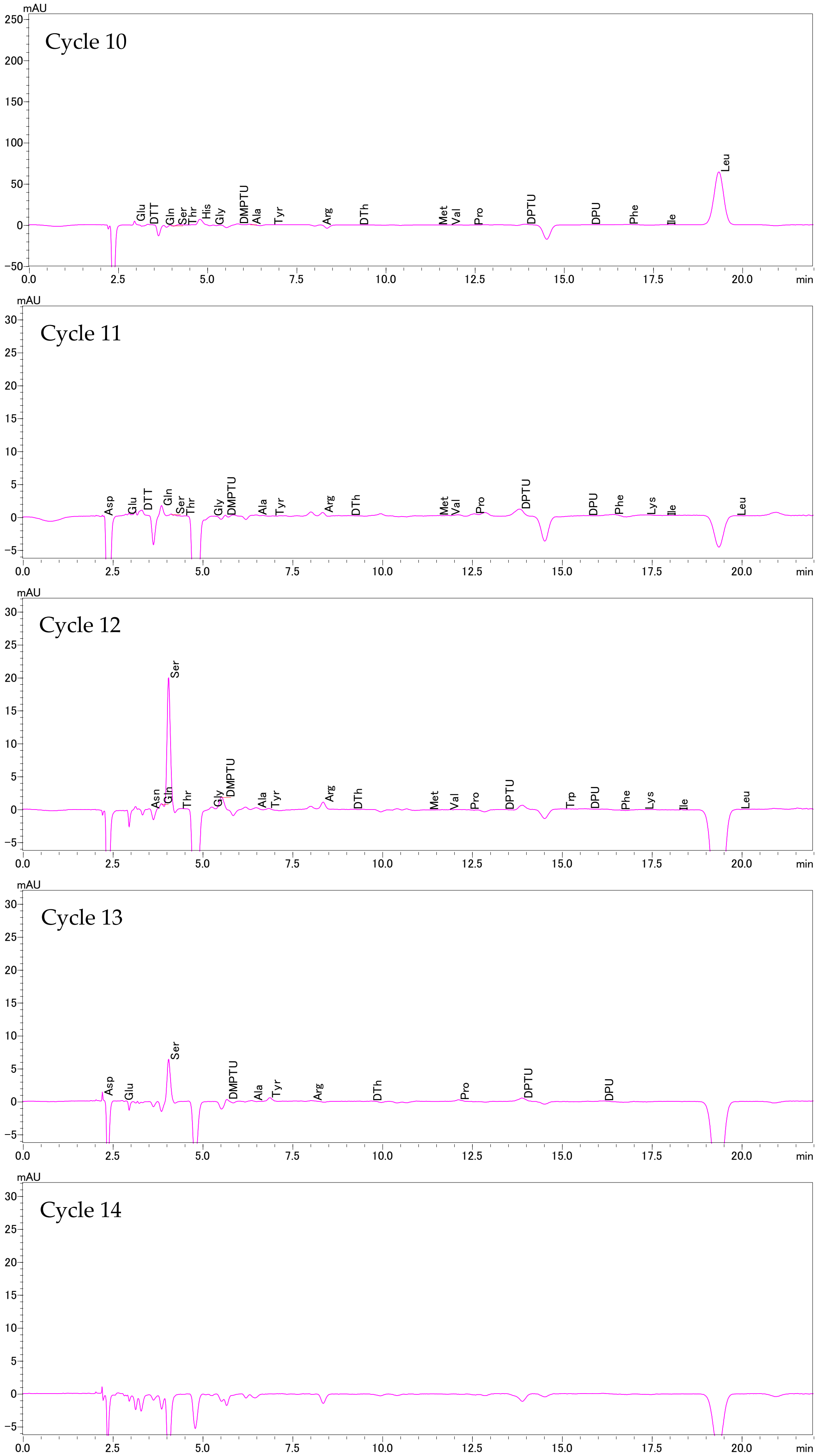

mAU
Cycle 10
Glu
DTT
Gln
Ser
Thr
His
Gly
DMPTU
Ala
Tyr
Arg
DTh
Met
Val
Pro
DPTU
DPU
Phe
Ile
Leu
min
mAU
Cycle 11
Asp
Glu
DTT
Gln
Ser
Thr
Gly
DMPTU
Ala
Tyr
Arg
DTh
Met
Val
Pro
DPTU
DPU
Phe
Lys
Ile
Leu
min
mAU
Cycle 12
Ser
Asn
Gln
Thr
Gly
DMPTU
Ala
Tyr
Arg
DTh
Met
Val
Pro
DPTU
Trp
DPU
Phe
Lys
Ile
Leu
min
mAU
Cycle 13
Asp
Glu
Ser
DMPTU
Ala
Tyr
Arg
DTh
Pro
DPTU
DPU
min
mAU
Cycle 14
min

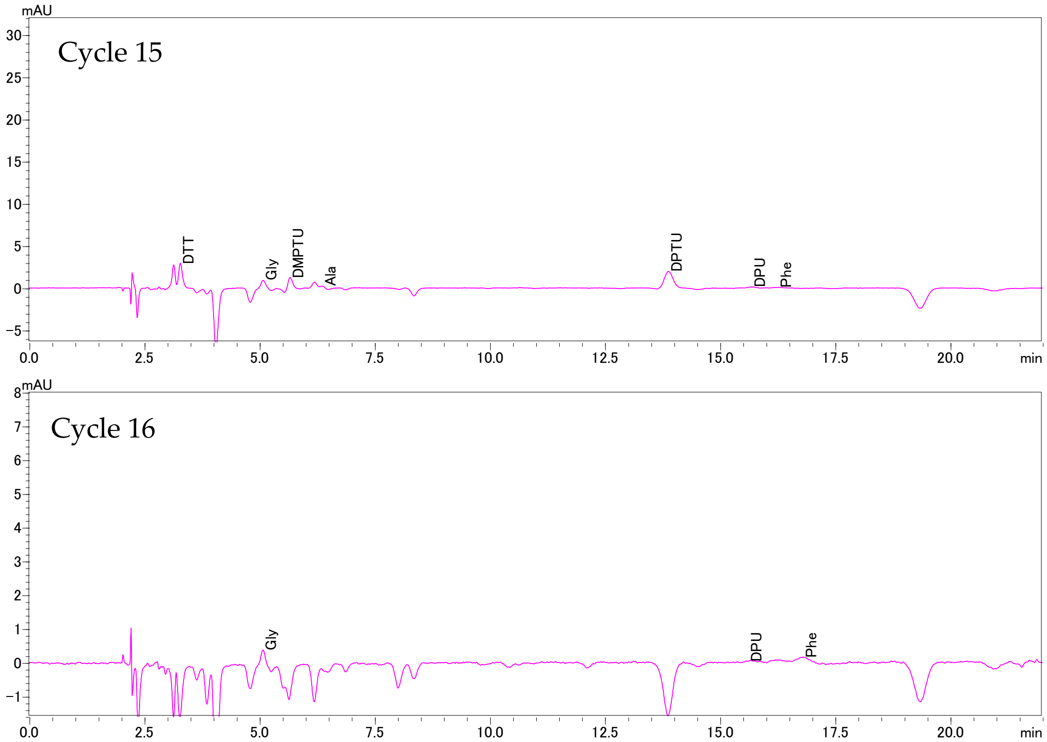

Figure S5. HPLC chromatograms of the amino acids released from each Edman degradation cycle of Mr3.8.

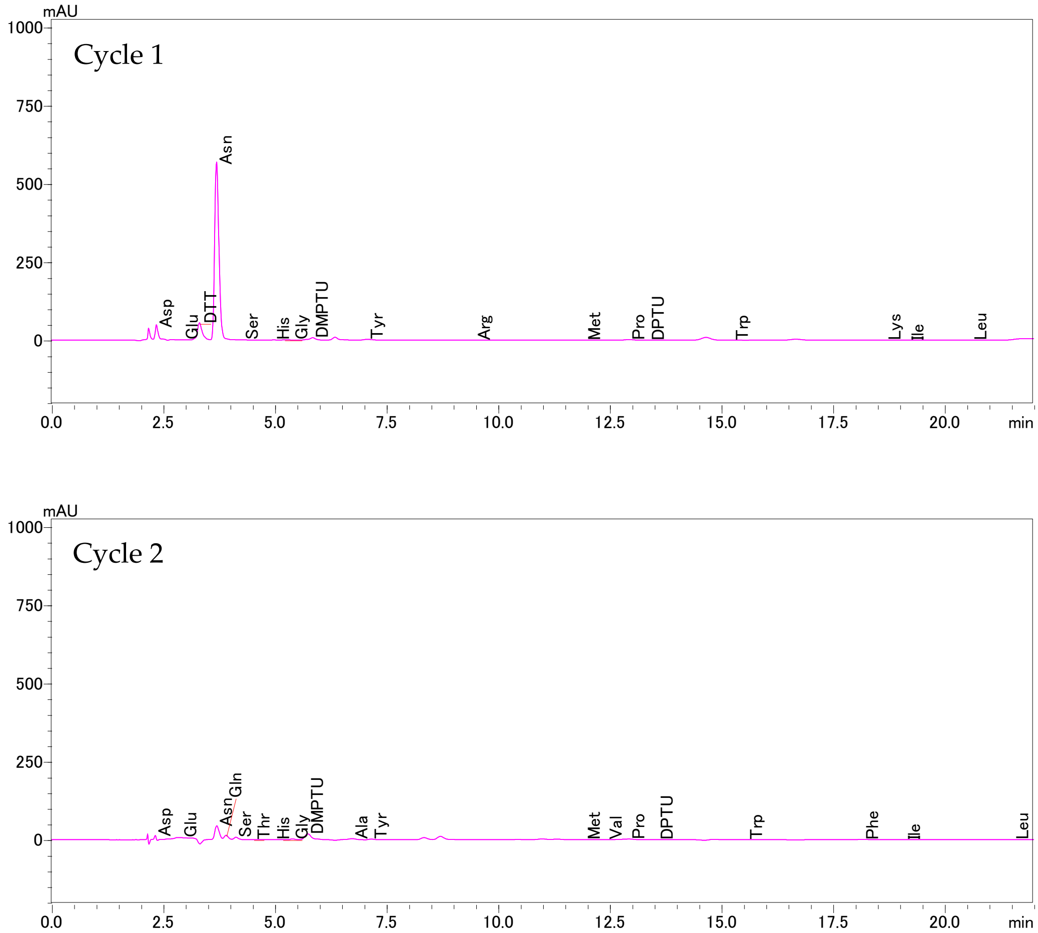

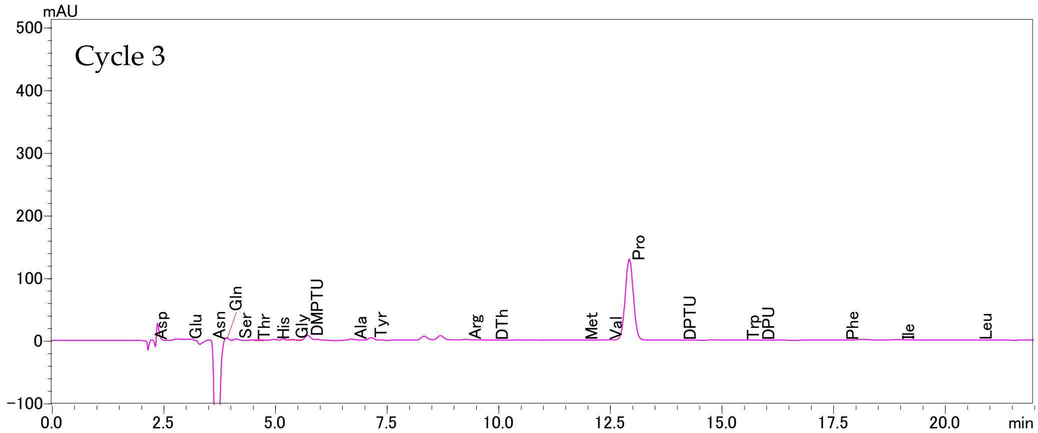
Cycle 3
mAU
500
400
300
200
100
0
−100
0.0
2.5
5.0
7.5
10.0
12.5
15.0
17.5
20.0
min
Asp
Glu
Asn
Gln
Ser
Thr
His
Gly
DMPTU
Ala
Tyr
Arg
DTh
Met
Val
Pro
DPTU
Trp
DPU
Phe
Ile
Leu

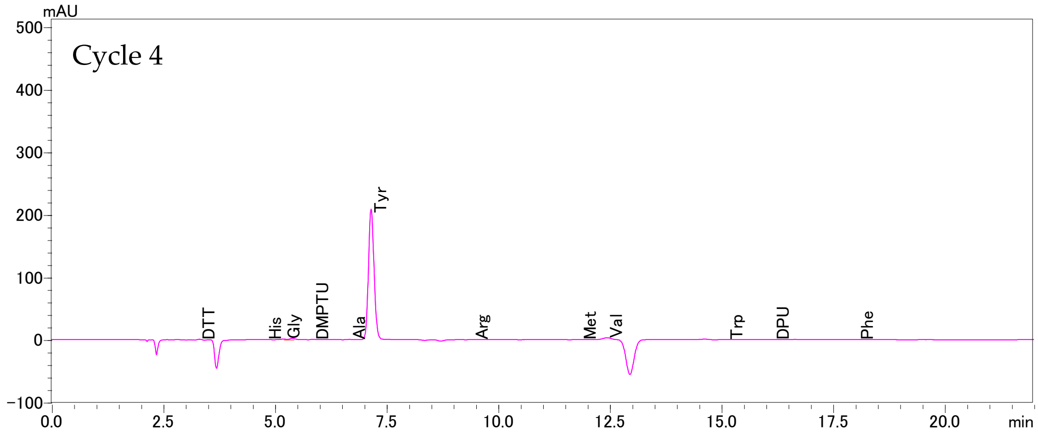
Cycle 4
mAU
500
400
300
200
100
0
−100
0.0
2.5
5.0
7.5
10.0
12.5
15.0
17.5
20.0
min
DTT
His
Gly
DMPTU
Ala
Tyr
Arg
Met
Val
Trp
DPU
Phe

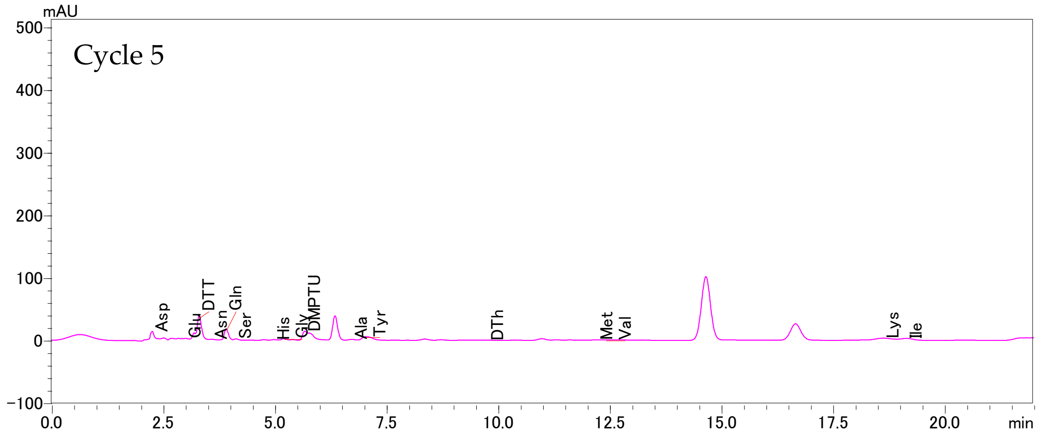
Cycle 5
mAU
500
400
300
200
100
0
−100
0.0
2.5
5.0
7.5
10.0
12.5
15.0
17.5
20.0
min
Asp
Glu
DTT
Asn
Gln
Ser
His
Gly
DMPTU
Ala
Tyr
DTh
Met
Val
Lys
Ile

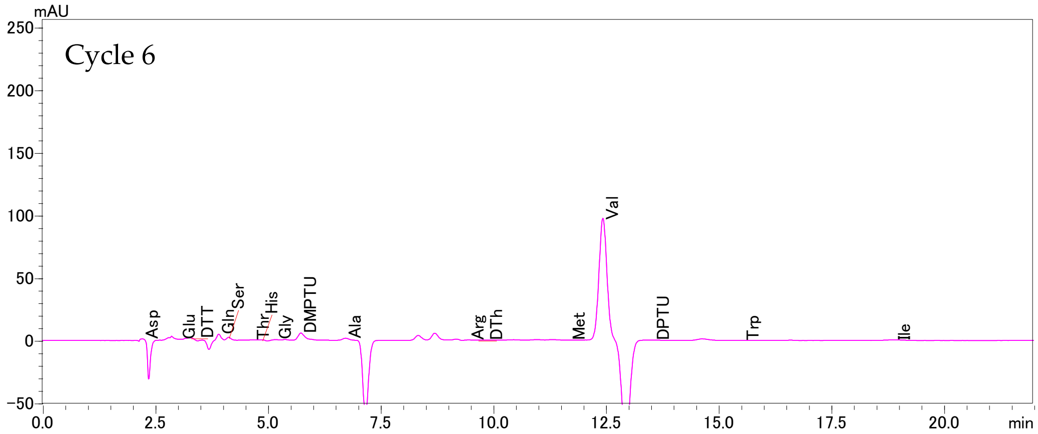
Cycle 6
mAU
250
200
150
100
50
0
−50
0.0
2.5
5.0
7.5
10.0
12.5
15.0
17.5
20.0
min
Asp
Glu
DTT
Gln
Ser
Thr
His
Gly
DMPTU
Ala
Arg
DTh
Met
Val
DPTU
Trp
Ile

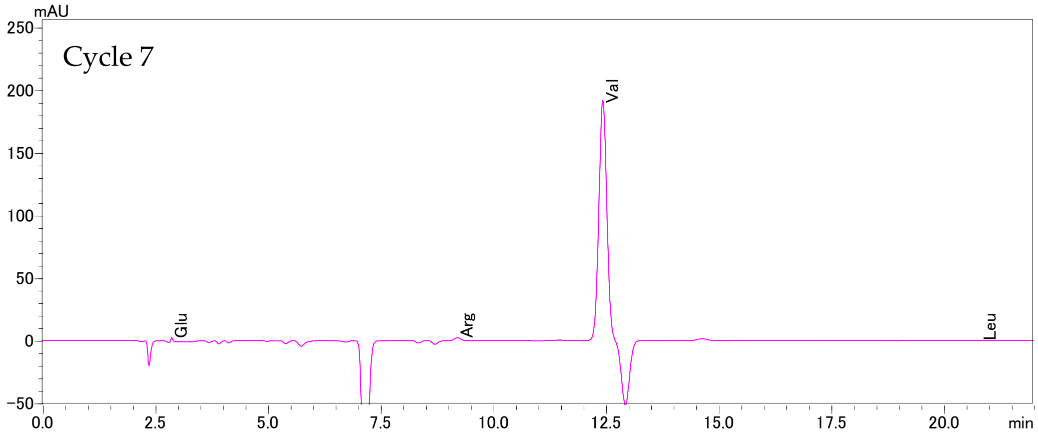
Cycle 7
mAU
250
200
150
100
50
0
−50
0.0
2.5
5.0
7.5
10.0
12.5
15.0
17.5
20.0
min
Glu
Arg
Val
Leu

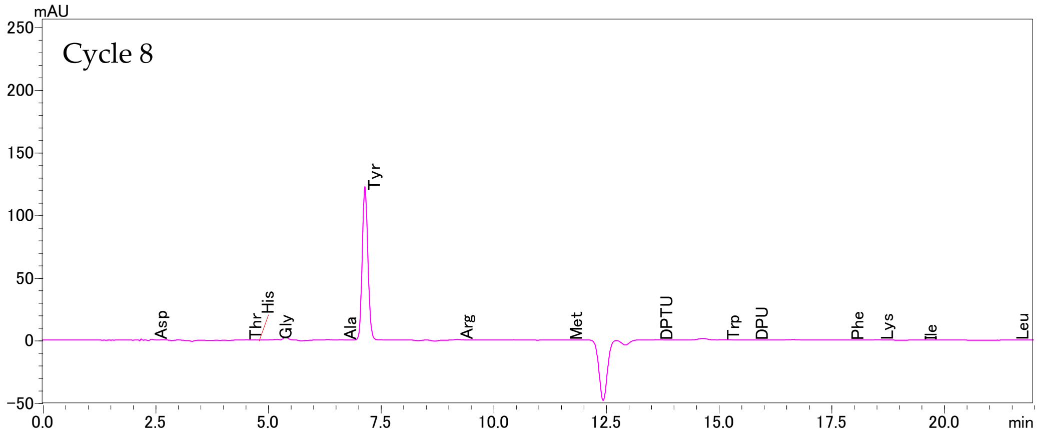
Cycle 8
mAU
250
200
150
100
50
0
−50
0.0
2.5
5.0
7.5
10.0
12.5
15.0
17.5
20.0
min
Asp
Thr
His
Gly
Ala
Tyr
Arg
Met
DPTU
Trp
DPU
Phe
Lys
Ile
Leu

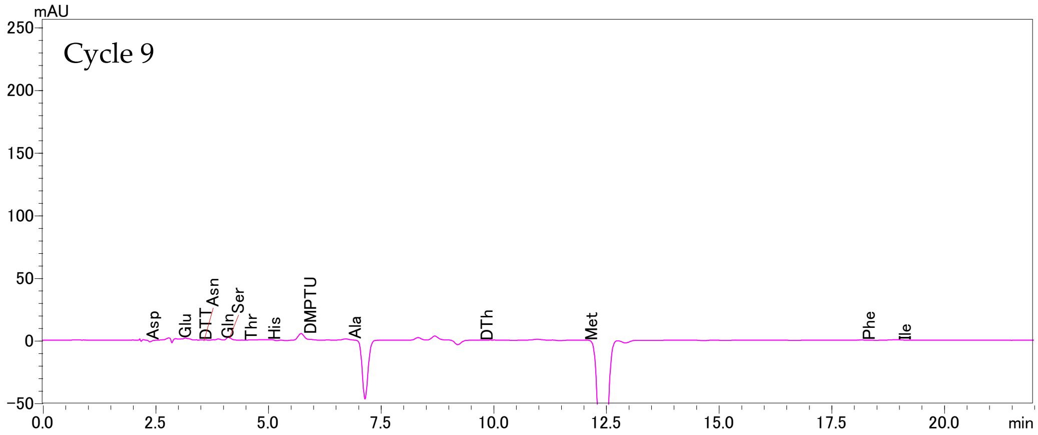
Cycle 9
mAU
250
200
150
100
50
0
−50
0.0
2.5
5.0
7.5
10.0
12.5
15.0
17.5
20.0
min
Asp
Glu
DTT
Asn
Gln
Ser
Thr
His
DMPTU
Ala
DTh
Met
Phe
Ile

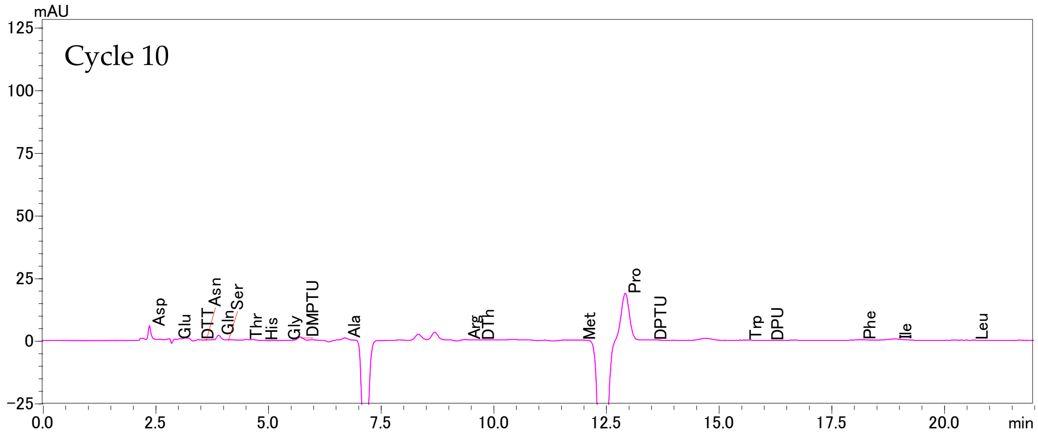
Cycle 10
mAU
125
100
75
50
25
0
−25
0.0
2.5
5.0
7.5
10.0
12.5
15.0
17.5
20.0
min
Asp
Glu
DTT
Asn
Gln
Ser
Thr
His
Gly
DMPTU
Ala
Arg
DTh
Met
Pro
DPTU
Trp
DPU
Phe
Ile
Leu

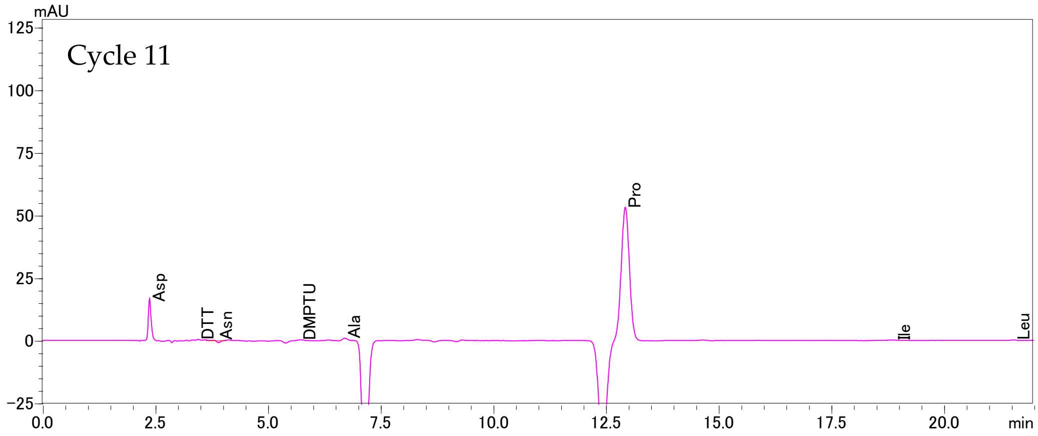

mAU
125
100
75
50
25
0
−25
Cycle 11
Asp
DTT
Asn
DMPTU
Ala
Pro
Ile
Leu
0.0
2.5
5.0
7.5
10.0
12.5
15.0
17.5
20.0
min

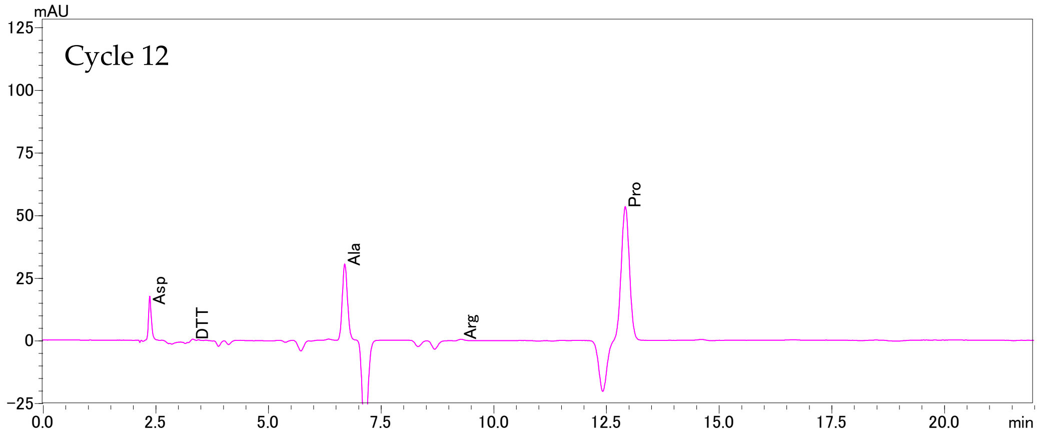

mAU
125
100
75
50
25
0
−25
Cycle 12
Asp
DTT
Ala
Arg
Pro
0.0
2.5
5.0
7.5
10.0
12.5
15.0
17.5
20.0
min

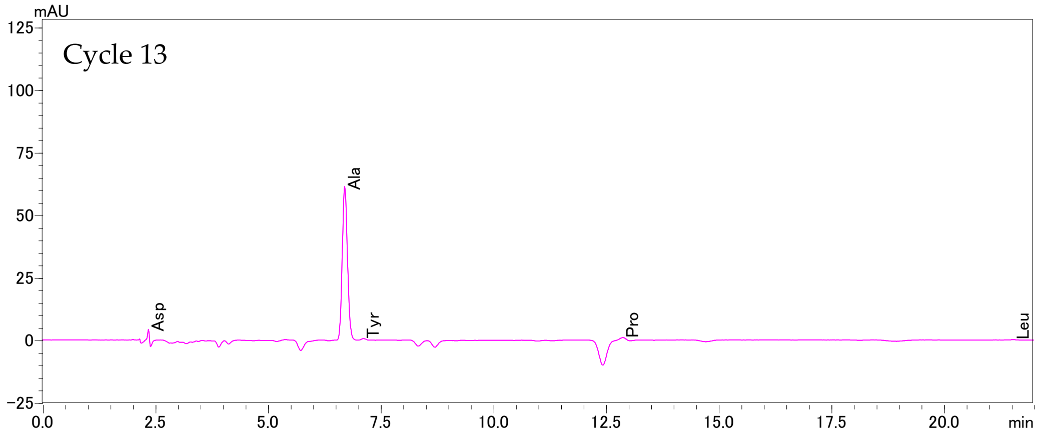

mAU
125
100
75
50
25
0
−25
Cycle 13
Asp
Ala
Tyr
Pro
Leu
0.0
2.5
5.0
7.5
10.0
12.5
15.0
17.5
20.0
min

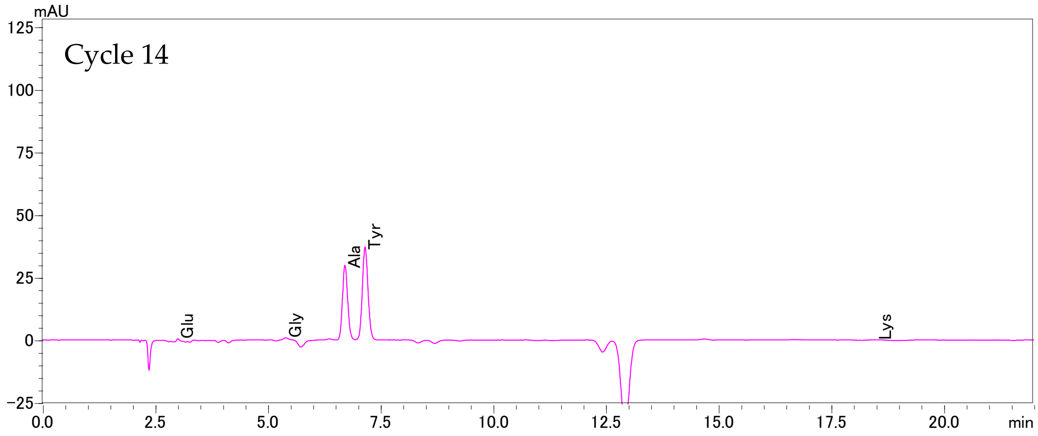

mAU
125
100
75
50
25
0
−25
Cycle 14
Glu
Gly
Ala
Tyr
Lys
0.0
2.5
5.0
7.5
10.0
12.5
15.0
17.5
20.0
min

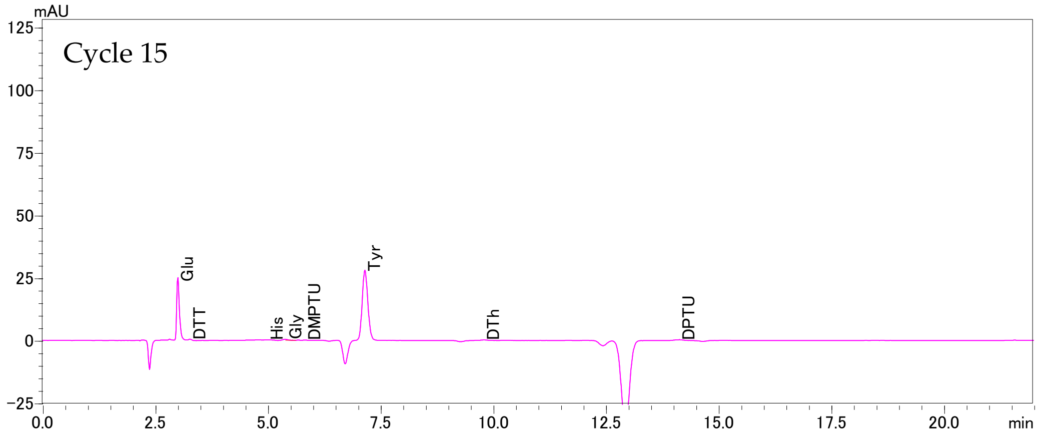
Cycle 15
mAU
125
100
75
50
25
0
−25
0.0
2.5
5.0
7.5
10.0
12.5
15.0
17.5
20.0
min
Glu
DTT
His
Gly
DMPTU
Tyr
DTh
DPTU

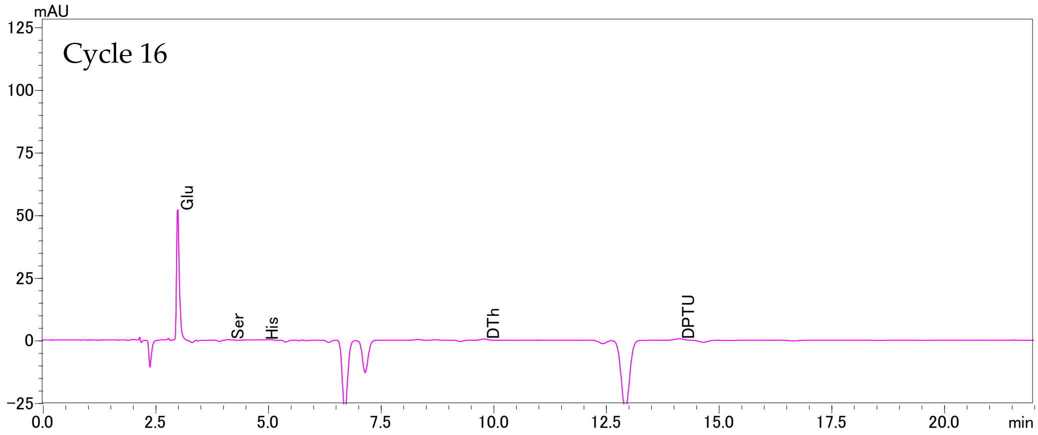
Cycle 16
mAU
125
100
75
50
25
0
−25
0.0
2.5
5.0
7.5
10.0
12.5
15.0
17.5
20.0
min
Glu
Ser
His
DTh
DPTU

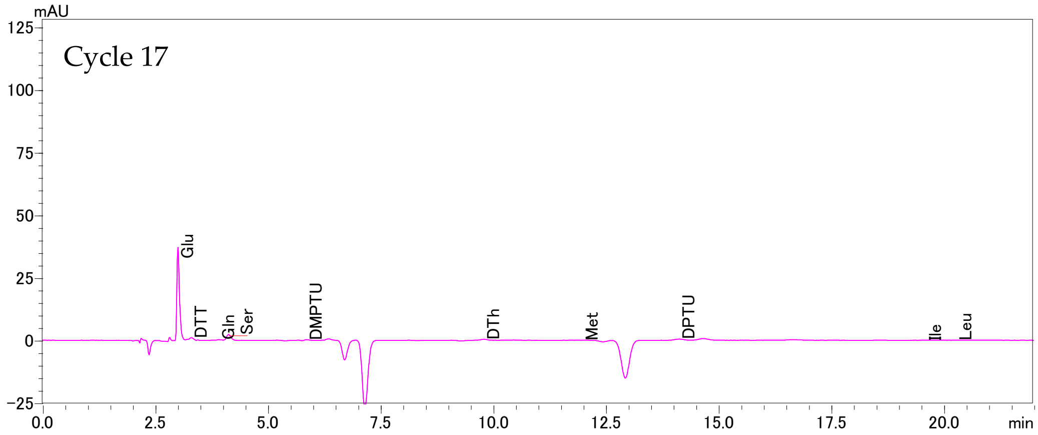
Cycle 17
mAU
125
100
75
50
25
0
−25
0.0
2.5
5.0
7.5
10.0
12.5
15.0
17.5
20.0
min
Glu
DTT
Gln
Ser
DMPTU
DTh
Met
DPTU
Ile
Leu

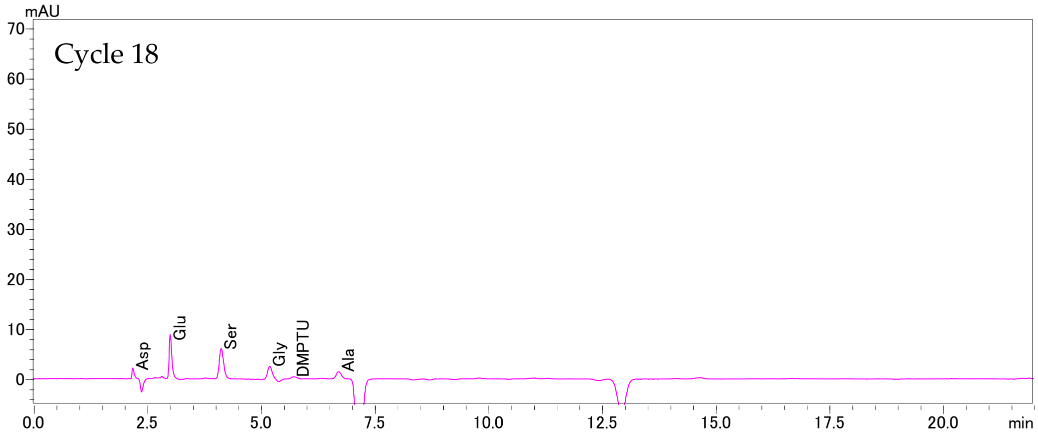
Cycle 18
mAU
70
60
50
40
30
20
10
0
0.0
2.5
5.0
7.5
10.0
12.5
15.0
17.5
20.0
min
Asp
Glu
Ser
Gly
DMPTU
Ala

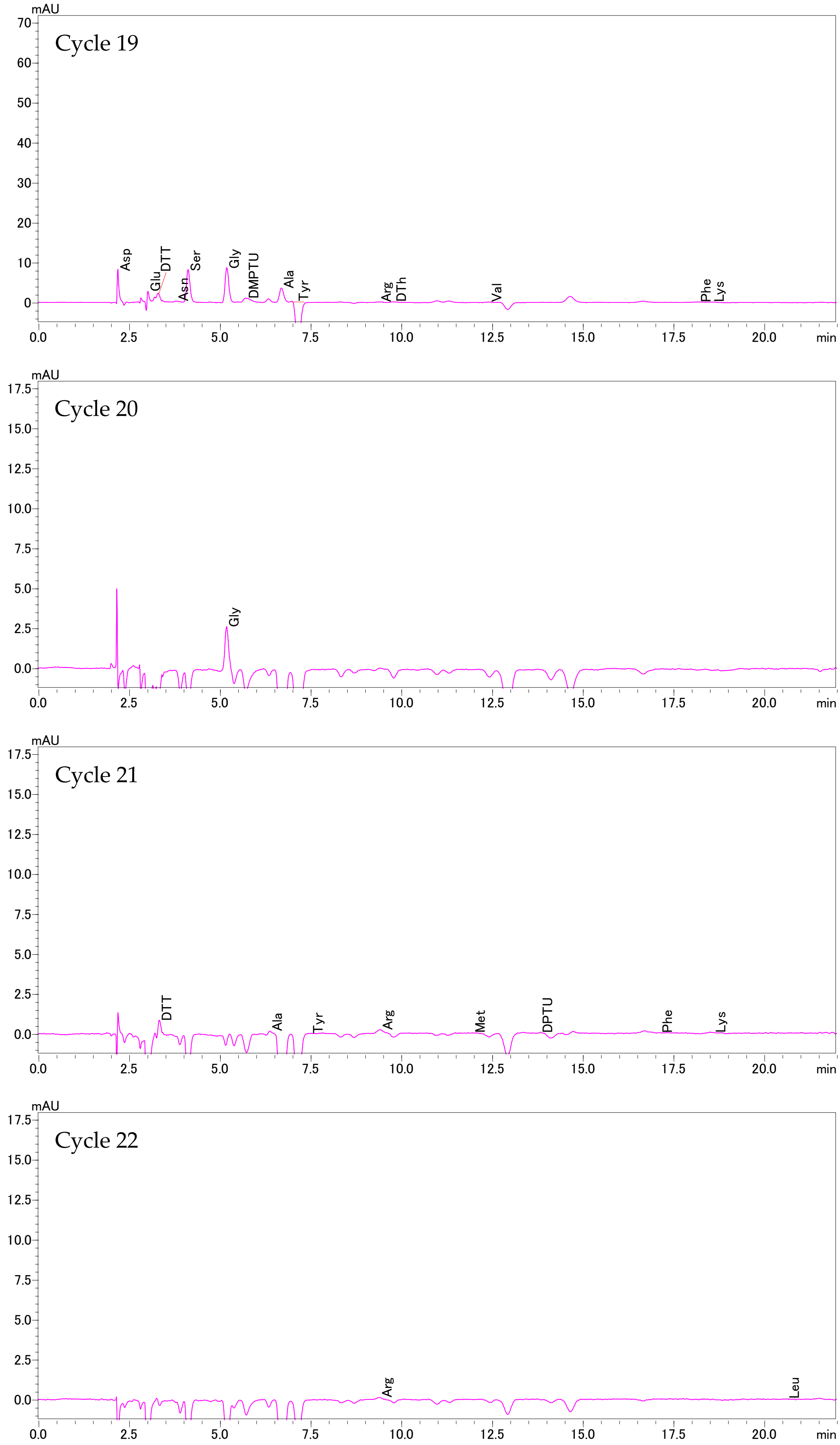

Figure S6. HPLC chromatograms of the amino acids released from each Edman degradation cycle of TCP.
